# Outcome measures for electric field modeling in tES and TMS: A systematic review and large-scale modeling study

**DOI:** 10.1016/j.neuroimage.2023.120379

**Published:** 2023-09-15

**Authors:** Sybren Van Hoornweder, Marten Nuyts, Joana Frieske, Stefanie Verstraelen, Raf L.J. Meesen, Kevin A. Caulfield

**Affiliations:** a REVAL - Rehabilitation Research Center, Faculty of Rehabilitation Sciences, University of Hasselt, Diepenbeek, Belgium; b Movement Control and Neuroplasticity Research Group, Department of Movement Sciences, Group Biomedical Sciences, KU Leuven, Leuven, Belgium; c Brain Stimulation Laboratory, Department of Psychiatry, Medical University of South Carolina, Charleston, SC, United States

**Keywords:** Electric field (E-field) modeling, Transcranial electrical stimulation (tES), Transcranial direct current stimulation (tDCS), Finite element method (FEM), Region of interest (ROI) analyses, Whole brain analyses

## Abstract

**Background:**

Electric field (E-field) modeling is a potent tool to estimate the amount of transcranial magnetic and electrical stimulation (TMS and tES, respectively) that reaches the cortex and to address the variable behavioral effects observed in the field. However, outcome measures used to quantify E-fields vary considerably and a thorough comparison is missing.

**Objectives:**

This two-part study aimed to examine the different outcome measures used to report on tES and TMS induced E-fields, including volume- and surface-level gray matter, region of interest (ROI), whole brain, geometrical, structural, and percentile-based approaches. The study aimed to guide future research in informed selection of appropriate outcome measures.

**Methods:**

Three electronic databases were searched for tES and/or TMS studies quantifying E-fields. The identified outcome measures were compared across volume- and surface-level E-field data in ten tES and TMS modalities targeting two common targets in 100 healthy individuals.

**Results:**

In the systematic review, we extracted 308 outcome measures from 202 studies that adopted either a gray matter volume-level (*n* = 197) or surface-level (*n* = 111) approach. Volume-level results focused on E-field magnitude, while surface-level data encompassed E-field magnitude (*n* = 64) and normal/tangential E-field components (*n* = 47). E-fields were extracted in ROIs, such as brain structures and shapes (spheres, hexahedra and cylinders), or the whole brain. Percentiles or mean values were mostly used to quantify E-fields. Our modeling study, which involved 1,000 E-field models and > 1,000,000 extracted E-field values, revealed that different outcome measures yielded distinct E-field values, analyzed different brain regions, and did not always exhibit strong correlations in the same within-subject E-field model.

**Conclusions:**

Outcome measure selection significantly impacts the locations and intensities of extracted E-field data in both tES and TMS E-field models. The suitability of different outcome measures depends on the target region, TMS/tES modality, individual anatomy, the analyzed E-field component and the research question. To enhance the quality, rigor, and reproducibility in the E-field modeling domain, we suggest standard reporting practices across studies and provide four recommendations.

## 1. Introduction

Electric field (E-field) modeling is a computational approach to estimate the amount of transcranial electrical stimulation (tES) and transcranial magnetic stimulation (TMS) that reaches the cortex (Thielscher et al., 2015; Huang et al., 2019). By segmenting an individual’s structural magnetic resonance imaging (MRI) scan into different tissue types such as skin, bone, cerebrospinal fluid (CSF), gray matter, and white matter, it is possible to simulate the E-fields induced by tES and TMS. By doing so, E-field modeling provides a potent tool to individually examine the effects of tES and TMS on the brain, and to investigate the high variability in efficacy that is currently observed across individuals. Previously, E-field modeling has enabled researchers to derive novel tES montages and identify dose-response relationships (Datta et al., 2009; Caulfield and George, 2022; Wischnewski et al., 2021; KA Caulfield et al., 2020; Suen et al., 2021; Kasten et al., 2019), has suggested optimal stimulation targets in clinical cohorts (KE Mantell et al., 2021; S Minjoli et al., 2017), and has pinpointed which cortical regions are likely being activated with different protocols (Weise et al., 2022). Software packages such as SimNIBS and ROAST have catalyzed the use of E-field modeling (Thielscher et al., 2015; Huang et al., 2019). Despite the standardization offered by these software packages, crucial experimental decisions made by researchers still vary considerably across studies. While the importance of factors such as MRI parameters (S Van Hoornweder et al., 2022; S Van Hoornweder et al., 2022; Nielsen et al., 2018; Huang et al., 2017), electrode placement accuracy (Opitz et al., 2018), head model detail (Weise et al., 2022; Huang et al., 2017; Khadka and Bikson, 2020) and meshing approach (Huang et al., 2019; Puonti et al., 2020) have been investigated, an often-overlooked determinant of E-field modeling results is the selected outcome measure.

Selection of an appropriate outcome measure to quantify E-fields is not trivial, as E-fields are complex vectorial fields that are influenced by factors such as the used TMS / tES modality and a person’s anatomy. To date, E-fields produced by tES and TMS have been quantified in many ways, yet a consensus about which feature to extract from E-field data, as well as how to extract it, is still absent. A thorough examination of outcome measures is critical as it directly affects the results. For instance, some studies suggest that the normal component is key while other studies point towards the importance of E-field magnitude (BBB Zhang et al., 2022; Aberra et al., 2020; Nandi et al., 2022; Rawji et al., 2018). Likewise, E-fields have been quantified on both the volume- and surface-level. Sometimes a region of interest (ROI) is used (e.g., the left primary motor cortex [M1]) (Nandi et al., 2022; Saturnino et al., 2021; Antonenko et al., 2019; SM Rampersad et al., 2014; Saturnino et al., 2015; H Zhang et al., 2022), whereas other times, a whole brain approach is used, not restricted to certain brain regions (Laakso et al., 2019; Saturnino et al., 2017; D Antonenko et al., 2021). Even within the same approach, methodological heterogeneity is pervasive. Research has quantified E-field magnitude within spherical ROIs of considerably different radii, ranging between 0.5 and 45 mm, and has also used other geometric shapes such as a cubical ROI (Nandi et al., 2022; Saturnino et al., 2021; Antonenko et al., 2019; SM Rampersad et al., 2014; Saturnino et al., 2015; H Zhang et al., 2022). Thus, while most outcome measures pursue the common goal of extracting an E-field value from an E-field model, they do so in fundamentally different ways, examine different regions, and study different components.

To date, there has been no systematic investigation of the impact of modeling outcome measure choice on E-field results. Critically, the choice of outcome measure affects all modeling approaches even as more advanced segmentation and meshing techniques emerge, making it a key consideration both now and in the future. As the focus shifts toward unraveling dose-response relationships associated with E-field magnitude (Turi et al., 2022; Alekseichuk et al., 2022; Wischnewski et al., 2021; Jamil et al., 2017; Chhatbar et al., 2016), it is crucial that we are comparing the same brain regions, levels, and components, necessitating a critical evaluation of outcome measures. Therefore, in this study, we set out to examine and compare the breadth and frequency of different outcome measures that have been used to quantify E-fields. In Part 1, we conducted a systematic literature review to identify and describe all the different outcome measures that have been used so far to quantify tES- and TMS-induced E-fields. In Part 2, we modeled the outcome measures identified in Part 1 in 100 healthy adults to elucidate the impact of outcome measure choice, and to further facilitate the interpretation of previous work and inform future research. As different tES and TMS modalities stimulate varying volumes of gray matter with varying focality, we simulated and compared outcome measures across ten tES and TMS montages targeting the motor and prefrontal cortices.

## 2. Methods

Section 2.1 informs on the methods used in the systematic review, which constitutes the first part of this study. In Section 2.2, we report the methods employed in the computational modeling, which constitutes the second part of this study. As the objective of the current study is to offer insights into the quantification of E-fields, the second part will build upon the first part.

### 2.1. Systematic review: eligibility, search strategy and extracted information

We consulted three electronic databases (PubMed, Scopus, and Web of Science) to examine how E-field magnitude is quantified. Studies were included if they adhered to the following eligibility criteria: (1) full-text availability; (2) written in English; (3) modeling of tES and/or TMS in humans; (4) quantifying the magnitude, normal, or tangential E-field component.

Studies were excluded if insufficient information was provided to reproduce the outcome measure procedure. Given the significant advances made in the modeling field in the past decade, we confined our literature search to 2013–2023, with the final search taking place on April 25, 2023. Our search keys are shown in Supplementary Materials 1.

### 2.2. Computational modeling

#### 2.2.1. Head model creation overview

All simulations were performed in SimNIBS v4.0.0 (Thielscher et al., 2015). To investigate how different outcome measures affect E-field quantification, T1w and T2w structural MRI scans from 100 participants from the Human Connectome Project dataset were retrieved (22–35 years, 50 females) (Van Essen et al., 2012). Through the SimNIBS – Charm pipeline, MRI scans were segmented and meshed into tetrahedral head models (Thielscher et al., 2015; Puonti et al., 2020). All models were visually inspected to confirm that there were no segmentation errors.

#### 2.2.2. Electric field models

We modeled 3 tES and 2 TMS modalities commonly used over EEG 10–20 positions C3 (approximating the precentral gyrus, i.e., the motor cortex) and F3 (approximating the middle frontal gyrus, i.e., the prefrontal cortex), for a total of 10 models per person as shown in Table 1. These montages and target regions were selected not only due to their widespread use in the field, but also to cover different montages and brain regions which will provide valuable insights into the generalizability of our findings. Standard conductivity values were used (Supplementary Materials 2). As the induced E-field values are linearly proportional to the stimulation intensity, multiplying and dividing the obtained E-field values is a simple heuristic to convert our results to other intensities. To make these values most easily translatable to other stimulation intensities via multiplication or division, we computed models at 1 mA (tES) and 1 A/s (TMS).

#### 2.2.3. Assessments of outcome measures

Following the systematic review (Cf., 3.1. Systematic Review Results), we analyzed the identified outcome measures in the 10 E-field simulations in all 100 participants (i.e., 1000 E-field models) (Okamoto et al., 2004). In total, we examined >1000,000 outcome measures spanning several domains: Volume-level outcome measures were analyzed in the subject-specific gray matter volume, while surface-level outcome measures were analyzed on the middle gray matter surface (i.e., the standard surface-level SimNIBS output). Consistent with the standard SimNIBS approach, mean E-field values represent average E-field values per volume (volume-level approach) or surface (surface-level approach). Volume-level ROI structure creation failed in 20 out of 1000 E-field models. Surface-level E-field data could not be analyzed in MATLAB in 105 out of 1000 E-field models. In both instances, all of the concerned E-field models were excluded from the associated analyses.

## 3. Results

Consistent with the methods, Section 3.1. outlines the first part of the study, which involves the systematic review. The subsequent sections, Sections 3.2 and 3.3., present the second part of the study, using computational modeling to delve into the outcome measures explored in the first part of the study.

### 3.1. Systematic review results

**Fig. 1.**
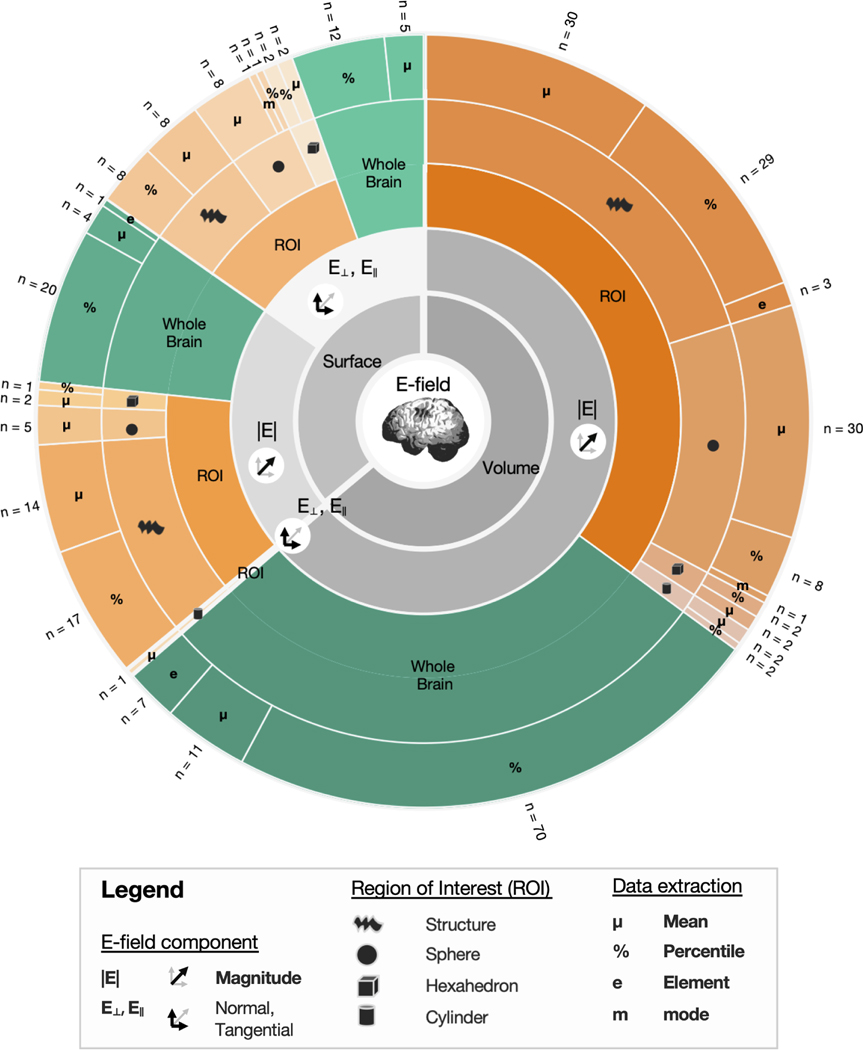
Outcome measures identified in the systematic review.

Our initial literature search resulted in the identification of 2214 studies. After removing 742 duplicates, and excluding 1276 articles based on title, abstract and full-text screening, 202 studies were included. The included studies quantified E-fields 308 times, with several studies reporting ≥ 2 outcome measures. Studies using several percentiles or the same ROI with varying sizes were considered as a single entry. Fig. 1 provides an overview of the used outcome measures. All measures were first categorized as volume- (*n* = 197) or surface-level data (*n* = 111) and further categorized into their respective subcategories. We first discuss volume-level outcome measures, followed by the surface-level outcome measures.

### 3.2. Volume-level outcome measures

The focus of Section 3.2. is to review and quantify volume-based outcome measures identified in the systematic review. E-fields were extracted on the volume-level 197 times, across 159 studies. These outcome measures either quantified E-fields in the whole brain (*n* = 88), or within an ROI (*n* = 109) (Table 2). All volumetric outcome measures reported E-field magnitude, except for a single study reporting the E-field normal component in a cylindrical ROI (SM Rampersad et al., 2014). As this study derived volume-level E-field data from surface-level data, by calculating the dot product of the E-field vector in the volume and the vector normal of the closest gray matter surface element, we will only discuss normal and tangential E-fields in Section 3.3.

The current section will cover Volume-level Whole Brain Percentile (Section 3.2.1.1.), Volume-level Whole Brain Mean and Element Wise (Section 3.2.1.2.), Volume-level Structural ROI (Section 3.2.2.1.), and Volume-level Geometrical ROI (Section 3.2.2.2.), finishing with a comparison of Volume-level Outcome Measures (Section 3.2.3.).

#### 3.2.1. Volume-level, whole brain outcome measures

Across 76 studies, 88 volumetric whole brain outcome measures were identified (Fig. 1). Of note, whole brain analyses are the only outcome measure on both the volume and surface-level that were used more by TMS studies than tES studies (Tables 2 and 3). This can likely be attributed to the focal nature of figure-of-eight TMS, which reduces the need to capture spatially-bound information.

##### 3.2.1.1. Volume-level, whole brain, percentile outcome measures.

The majority of the volume-level, whole brain outcome measures extracted a percentile (*n* = 70). Percentiles of 0, 25, 50, 75, 80, 90, 95, 98, 99, 99.5, 99.9, 99.99, and 100 % were used.

**Fig. 2.**
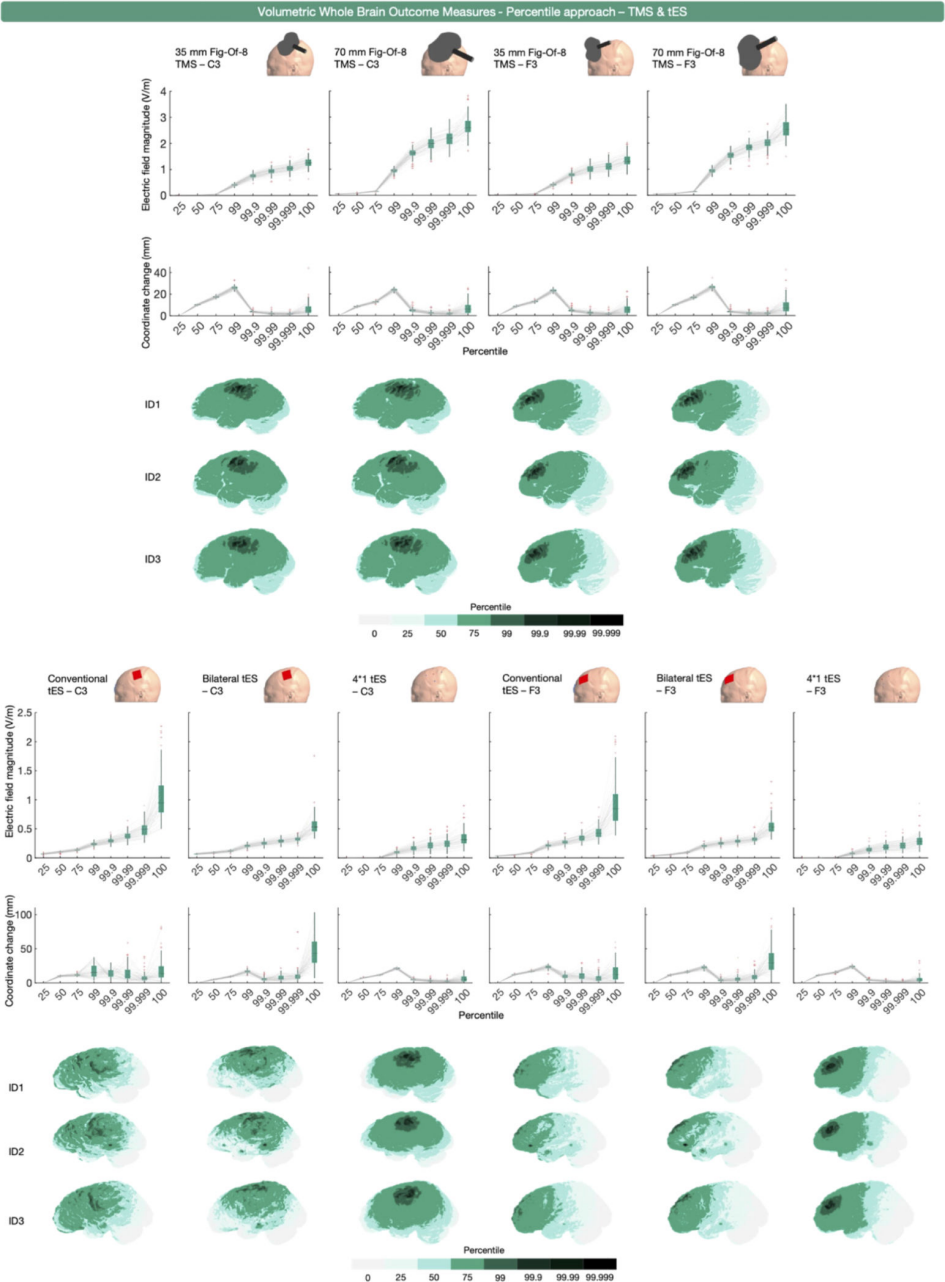
Volume-level, whole brain, percentile-based outcome measure for TMS (upper half) and tES (lower half) over C3 and F3. The upper row of boxplots shows E- field magnitude values obtained for different percentiles. The middle row of boxplots presents the mean coordinate position change of the tetrahedra associated with each percentile, compared to the previous one. The sharp drop in distance change (mm) with increasing percentiles starting from the 99th percentile can be attributed to the tetrahedra associated to the upper percentiles (> 99%) being numerically close compared to the tetrahedra associated to the lower percentiles. Collectively, these data indicate that the 100th percentile contains erroneous values (i.e., greater variability in E-field magnitude and coordinate change than similar iterative changes such as 99.99th to 99.999th percentiles). The volumes analyzed per percentile and modality are shown for 3 3 representative participants.

To investigate the utility of this approach, we extracted percentiles from the whole gray matter volume per participant and montage that were representative of this range (i.e., the 0, 25, 50, 75, 99, 99.9. 99.99. 99.999, and 100 % values). Fig. 2 shows the results for TMS and tES, respectively. Given that 45 studies reported the 100th percentile values, we particularly sought to elucidate whether there are substantial differences between other high percentile values (i.e., the 99.999th percentile vs. 100th percentile) which would inform on the suitability of comparing these studies.

The E-field magnitudes obtained by percentiles 25 to 75 were low compared to percentiles 90 to 100 for focal montages (i.e., 35 mm and 70 mm TMS and 4*1 tES). For non-focal montages (i.e., conventional and bilateral tES), the E-field magnitude increase per quantified percentile was much less steep, highlighting the inherently diffuse nature of these montages.

Regarding the differences between higher percentiles, there were substantial E-field magnitude differences between the 99.999th and 100th percentile approaches that were less apparent than going from the 99.99th to 99.999th percentile, highlighting the potential risks of utilizing a 100th percentile threshold. The mean increase in magnitude from percentile 99.999 to 100 ranged from 19.86 ± 8.39 % (35 mm figure-of-eight TMS over C3) to 110.74 ± 13.60 % (conventional tES over C3) whereas the increase from percentile 99.99 to 99.999 had a more restricted change in magnitude range between 11.16 ± 2.27 % (70 mm figure-eight TMS over C3) and 30.51 ± 8.18 % (conventional tES over C3). Also, we found that there were more substantial deviations in the mean position of the examined E-fields between the 99.999th and 100th percentiles. In terms of position, the highest observed mean deviation was a 45.88 ± 22.18 mm change (bilateral tES over C3). In contrast, the highest change in mean position from the 99.99th to 99.999th percentile was only 10.36 ± 8.87 mm (bilateral tES over C3). Put together, both the E-field magnitude and the associated mean tetrahedra position corroborate that the 100th percentile reflects an erroneous value (M Soldati and Laakso, 2020). Despite the perils of using the 100th percentile, 45 studies reported the 100th percentile E-field magnitude either in combination, in isolation, or reported the peak E-field magnitude without specifying what the peak represented (SM Rampersad et al., 2014; Alam et al., 2016; Amani et al., 2021; Ashikhmin and Aliev, 2016; Chung et al., 2022; Handiru et al., 2021; Jiang et al., 2020; Kenville et al., 2020; Khorrampanah et al., 2020; Klaus and Schutter, 2018; Miranda et al., 2016; Scheldrup et al., 2014; Shahid et al., 2014; C Thomas et al., 2019; Wang et al., 2021; Woods et al., 2015; Lu and Ueno, 2015; OF Afuwape et al., 2022; OF Afuwape et al., 2021; OF Afuwape et al., 2021; OF Afuwape et al., 2022; S Fiocchi et al., 2016; J Gomez-Tames et al., 2019; Lee et al., 2016; Lewis et al., 2023; CS Li et al., 2019; Rastogi et al., 2017; Rastogi et al., 2018; M Soldati and Laakso, 2020; F Syeda et al., 2017; F Syeda et al., 2017; F Syeda et al., 2017; Tzirini et al., 2021; OF Afuwape et al., 2021; Bortoletto et al., 2016; Csifcsák et al., 2018; Cvetkovíc et al., 2015; Cvetkovic et al., 2023; Kessler et al., 2013; Laakso et al., 2015; Metwally et al., 2015; Miranda et al., 2013; N Mittal et al., 2022; N Mittal et al., 2022; Splittgerber et al., 2020; Zmeykina et al., 2020). While the majority of these studies reported multiple percentiles or did not describe what the peak represented, this finding highlights that the field needs a better understanding of what E-field measures reflect, in addition to better and more standardized reporting conventions. Consistent with these findings, for all remaining outcome measures discussed in the current work, except for the percentile outcome measures, values > 99.999th percentile were replaced with the 99.999th percentile value.

Fig. 2 also highlight an important characteristic of whole brain, percentile-based E-field extraction. These analyses do not rely on spatial assumptions, thereby reducing the likelihood of overlooking brain regions that may have received physiologically relevant stimulation. This is particularly compelling when assuming a magnitude-centric dose-response relationship for TMS and tES where regions that are exposed to stronger E-fields may show stronger physiological effects of stimulation, and for safety studies aiming to determine the maximum exposure to E- fields induced by these modalities. However, the spatial agnosticism inherent to percentile-based outcome measures comes with the trade-off of spatial uncertainty. For less focal types of stimulation such as conventional tES, the regions identified by the percentile-based whole brain approach may not necessarily align with the regions where maximal stimulation is expected. Additionally, for both focal and non-focal types of stimulation, the percentile approach can result in different brain areas being analyzed across persons due to inter-individual anatomical differences. This introduces the risk that specific percentile cutoffs may report E-fields in biologically relevant or irrelevant regions in a person-specific manner.

In summary, volume-based whole brain percentile outcome measures are commonly used in the literature, particularly for figure-of-eight TMS. A wide range of percentiles have previously been used, spanning from the 0th to 100th percentile. In general, more focal types of stimulation such as figure-of-eight TMS and 4*1 tES focus maximal stimulation underneath the center of the coil or center electrode, making the percentile reported more accurately reflect the E-field magnitude at the stimulation target. In contrast, percentile approaches for more diffuse methods of stimulation such as conventional and bilateral tES capture off-target effects midway between electrodes and report the E- field magnitudes of off-target effects. Here, we further compared the similarity between small percentile changes commonly used in the literature, such as iterative changes between the 99.9th, 99.99th, 99.999th, and 100th percentiles. Despite the 100th percentile being widely used, there are substantial differences in both the magnitude and locations analyzed of up to 110.74 % and 45.88 mm respectively compared to the 99.999th percentile. These data highlight the importance of outcome measure selection even within a modality, with small percentile changes corresponding to large differences in E-field magnitude and location analyzed. In the next section, we further examined the influence of outcome measure on E-field modeling findings by focusing on a whole brain mean E-field approach previously used in the literature.

##### 3.2.1.2. Volume-level, whole brain, mean and element-wise outcome measures.

**Fig. 3.**
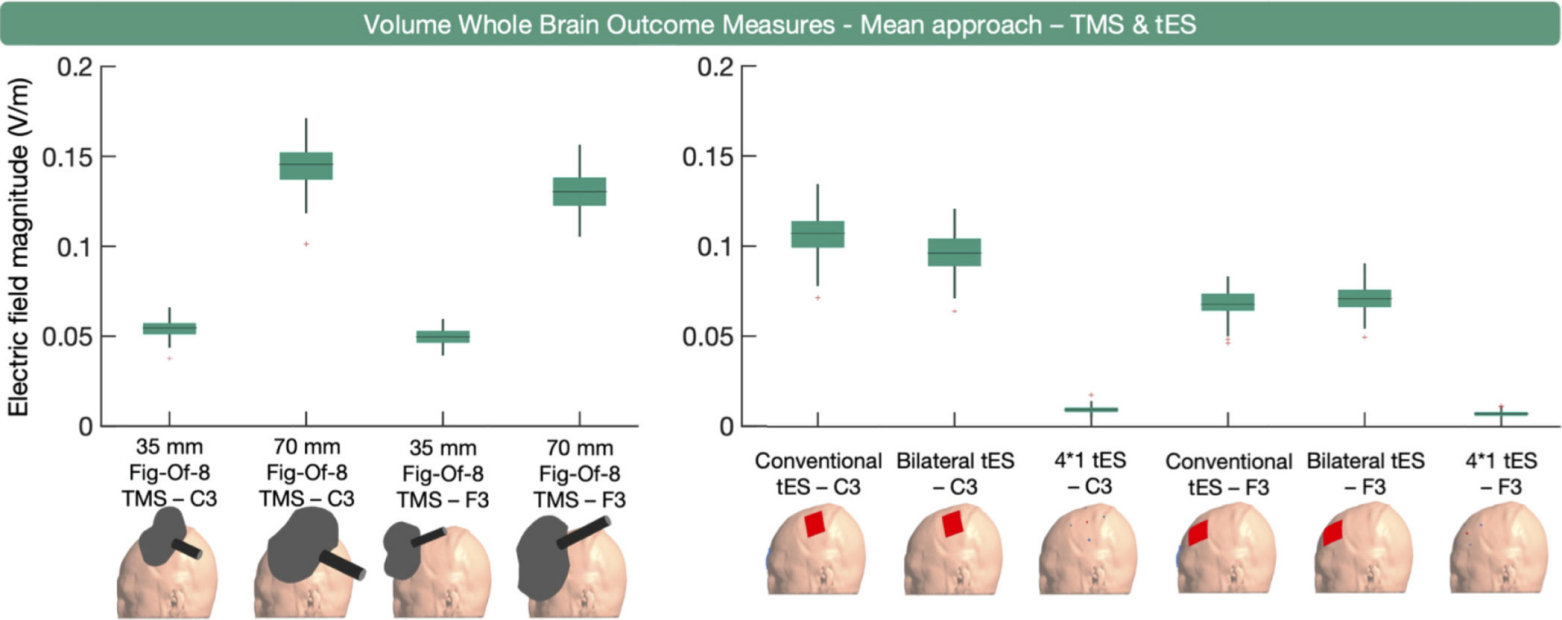
Volume-level, whole brain, mean E-field magnitude outcome measure per modality. On the left, the boxplots for TMS indicate that the larger 70 mm figure- eight TMS coil induces the highest E-field magnitude. On the right, the boxplots for tES indicate that conventional and bilateral tES induce substantially higher mean E-field magnitudes than 4 × 1 tES. Overall, the mean E-field magnitudes are higher when targeting C3 compared to F3.

Mean E-field magnitude in the whole brain was used as an outcome measure 11 times (Fig. 1). Beyond providing an overview of the general exposure to TMS and tES, this outcome measure has limited use. In contrast to the volume-level whole brain percentile approach discussed in the previous section being informative for more focal types of TMS and tES (e.g., 4*1 tES), where higher percentiles closely align with the targeted regions, whole brain mean outcome measures are uninformative for these focal modalities, as shown in Fig. 3. Some other drawbacks of the mean approach are that it is prone to outliers, such as the 100th percentile (which was not the case here as we replaced the 100th percentile by the 99.999th percentile, in line with Section 3.2.1.1.), and is influenced by the gray matter volume with larger volume potentially corresponding to lower mean values.

Seven outcome measures extracted E-field magnitude in an element-wise manner. These outcome measures extracted the mean of the highest elements (e.g., voxels, tetrahedra) in the E-field model, ranging from the 50, 100, 1000, or 10,000 highest elements. While element-wise extraction shares some resemblance with percentile-wise extraction, it has several drawbacks. It is biased by the spatial resolution of the head model (e.g., the number of tetrahedra in SimNIBS). Also, similar to the 100th percentile E-field magnitude, extracting the highest elements of a model implies that erroneous values are likely included.

In sum, whole brain mean and element-wise extraction are somewhat uncommon approaches relative to percentile-based approaches and can be prone to the inclusion of erroneously high values.

#### 3.2.2. Volume-level, region of interest outcome measures

The majority of the volume-level outcome measures (109 outcome measures across 97 studies) extracted E-fields within an ROI. Of these outcome measures, 31 related to TMS and 78 to tES. While the spatial confinement of ROIs can be advantageous to answer specific hypotheses, it can also be a drawback as it implies that potentially relevant E-fields outside the ROI are neglected, which holds particular relevance for non- focal modalities. Notably, the drawback of not including peak E-fields depends entirely on the research objectives of a study. When researchers have a specific spatially bound hypothesis, it can be valid to not include peak E-fields elsewhere, even if these are relevant from a neurophysiological point-of-view.

##### 3.2.2.1. Volume-level, structural region of interest outcome measures.

A structural, volume-level, ROI was used in 62 instances (Fig. 1). Volumetric structural ROIs offer flexibility, as they can be construed based on anatomical atlases and/or neuroimaging data. They are also individualized in a sense that they take brain anatomy and size into consideration. However, the transferability of these ROIs to other studies depends on whether or not a similar ROI was used and ROI volumes can differ across persons.

The majority of outcome measures extracted the mean E-field magnitude (*n* = 30) or a percentile E-field magnitude (*n* = 29) from structural ROIs. A smaller number of outcome measures (*n* = 3) used element-wise extraction. When using the percentile approach within a structural ROI, various percentiles were used either in isolation or combined, including the 0th, 25th, 50th, 75th, 80th, 95th, 99th, 99.5th, and 100th percentile. Notably, 17 outcome measures reported either the 100th percentile in isolation, combined, or did not describe what percentile reflected the peak E-field (Shahid et al., 2014; S Fiocchi et al., 2016; Benussi et al., 2021; Benussi et al., 2022; Suzuki et al., 2022; C Thomas et al., 2019; DaSilva et al., 2015; Datta et al., 2013; Edwards et al., 2013; WH Lee et al., 2018; Lu and Ueno, 2017; M Parazzini et al., 2017; Parazzini et al., 2015; Passera et al., 2022; Rezaee et al., 2020; Seibt et al., 2015; Thomas et al., 2018). As previously discussed and shown in Fig. 2, the use of the 100th percentile should be avoided due to widely varying ranges in magnitude and locations of maximal E-fields.

Drawing general conclusions about structural ROIs is challenging due to the infinite variability that is theoretically possible in their shapes and sizes. Here, we used the left precentral gyrus and left middle frontal gyrus as structural ROIs for TMS and tES targeting C3 and F3, respectively. Both structural ROIs were defined via the Harvard-Oxford Cortical Atlas. Specifically, for each participant we transformed the subject-space volumetric E-field data to MNI space using FSL – FLIRT with standard parameters. The inverse transformation matrix obtained from doing so was then used to transform the FSL – Harvard-Oxford Cortical Atlas to subject space. Via nii2mesh (SimNIBS) and custom MATLAB code, the subject-space structural volume-level ROIs were used to extract mean E-field magnitude and the 25th, 50th, 75th and 99.999th percentiles per participant.

**Fig. 4.**
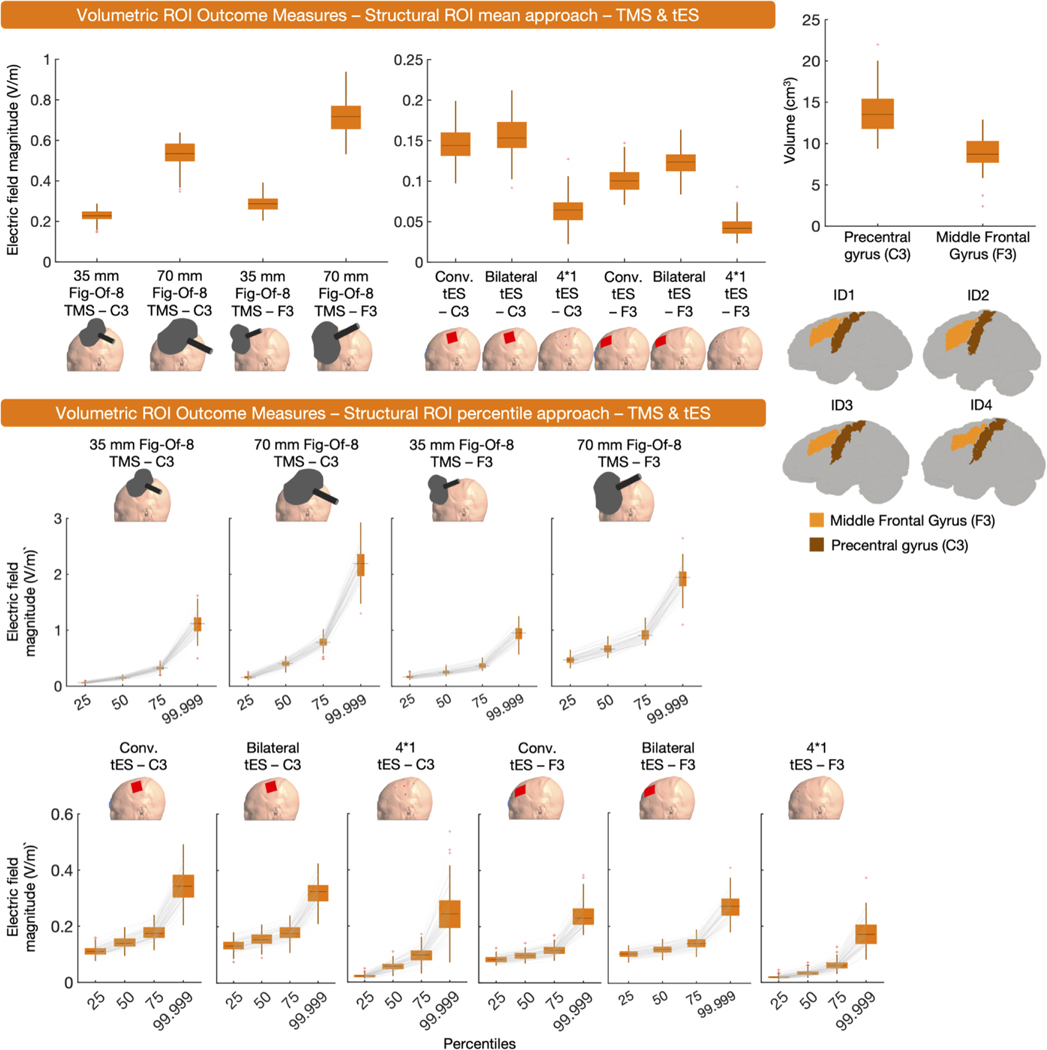
Volume-level, structural ROI, mean and percentile outcome measures for TMS and tES. In the upper right corner, the volume of each ROI and the ROIs in four representative participants are shown. The upper panel presents the mean E-field magnitude induced by TMS and tES in the structural ROI, while the lower panel shows percentile E-field magnitude values obtained from the ROIs for both modalities. 70 mm figure-eight TMS consistently resulted in higher E-field magnitudes, compared to 35 mm figure-of-eight TMS. Bilateral tES induced the highest E-field magnitudes in the ROIs of all tES modalities.

Overall, the mean and percentile values shown in Fig. 4 indicate that E-field magnitudes are mostly larger in the precentral gyrus, as a result of C3 TMS/tES, compared to the middle frontal gyrus, as a result of F3 TMS/tES. This observation corroborates the whole brain analyses in that C3 TMS/tES results in larger E-field magnitudes than F3 TMS/tES (Figs. 2 and 3). The only exception to this is the mean E-field magnitude induced in the structural ROIs as a result of TMS, with larger magnitudes in the F3 ROI, compared to the C3 ROI.

Here, we additionally found that bilateral tES induced the highest E- field magnitudes in the ROIs in comparison to other forms of tES (i.e., conventional tES and 4*1 tES) (Fig. 4, upper panel). This contrasts the whole brain analyses, where conventional tES induced the largest E-field magnitudes. This discrepancy elegantly illustrates how different outcome measures offer different yet complementary insights into E-fields.

In sum, volume-based structural ROIs are the most common outcome measure and can be used to identify the E-field magnitude within specific cortical targets. As the researcher chooses the ROI location based on an atlas or structural/functional neuroimaging data, there is flexibility in this approach. However, in the more diffuse conventional or bilateral tES modalities, defining the structural location immediately under the electrodes may miss the region of maximal stimulation due to the properties of the tES montage which can be a limitation if one is ignorant of this and/or assumes the region receiving peak stimulation to be physiologically relevant. Thus, structural ROIs and whole brain percentiles are complementary in their ability to describe both spatially restricted and whole brain maximal E-fields.

##### 3.2.2.2. Volume-level, geometrical region of interest outcome measures.

Thirty-nine studies used a spherical ROI to extract mean E-field magnitude (Fig. 1). The mean (*n* = 30), a percentile (*n* = 8), or the mode (*n* = 1) were extracted. Spherical ROI sizes varied considerably across studies, with radii of 0.5, 3, 5, 10, 15, 20, 25, and 45 mm being used. Apart from spherical ROIs, other shapes such as hexahedra (*n* = 4) and cylinders (*n* = 3) were used.

Geometrical ROIs are easy to implement and they ensure that approximately the same volume is analyzed across individuals and/or regions, with slight variations potentially occurring due to the included volumes typically being confined to a tissue of interest. However, their spatial rigidity implies that the ROI is not individualized in an anatomical sense, which can be achieved via structural ROIs.

We used spherical, hexahedral and cylindrical ROIs, centered at the subject-space transformed MNI coordinates of the cortical projections of F3 (MNI coordinate = − 35.5, 49.4, 32.4) and C3 (MNI coordinate = − 52.2, − 16.4, 57.8), depending on the target site. As with all other analyses, the ROIs were restricted to only extract E-field values within the gray matter. For each shape, we used 8 ROIs. In the spherical and cylindrical ROIs, we used radii of 0.5, 1, 2.5, 5, 10, 20, 30, and 50 mm. Cylindrical ROIs were oriented with the central vector passing through the center of the head (MNI coordinate = 0 0 0, transformed to subject space) and the subject-space transformed MNI coordinate of F3 or C3, depending on the montage. This way, they considered the whole gray matter in terms of depth, which is also why they are typically used in literature. As for the hexahedral ROIs, they were positioned along the X, Y, and Z axes, with their volume adjusted to match the volume of the cylindrical ROIs for each radius size.

**Fig. 5.**
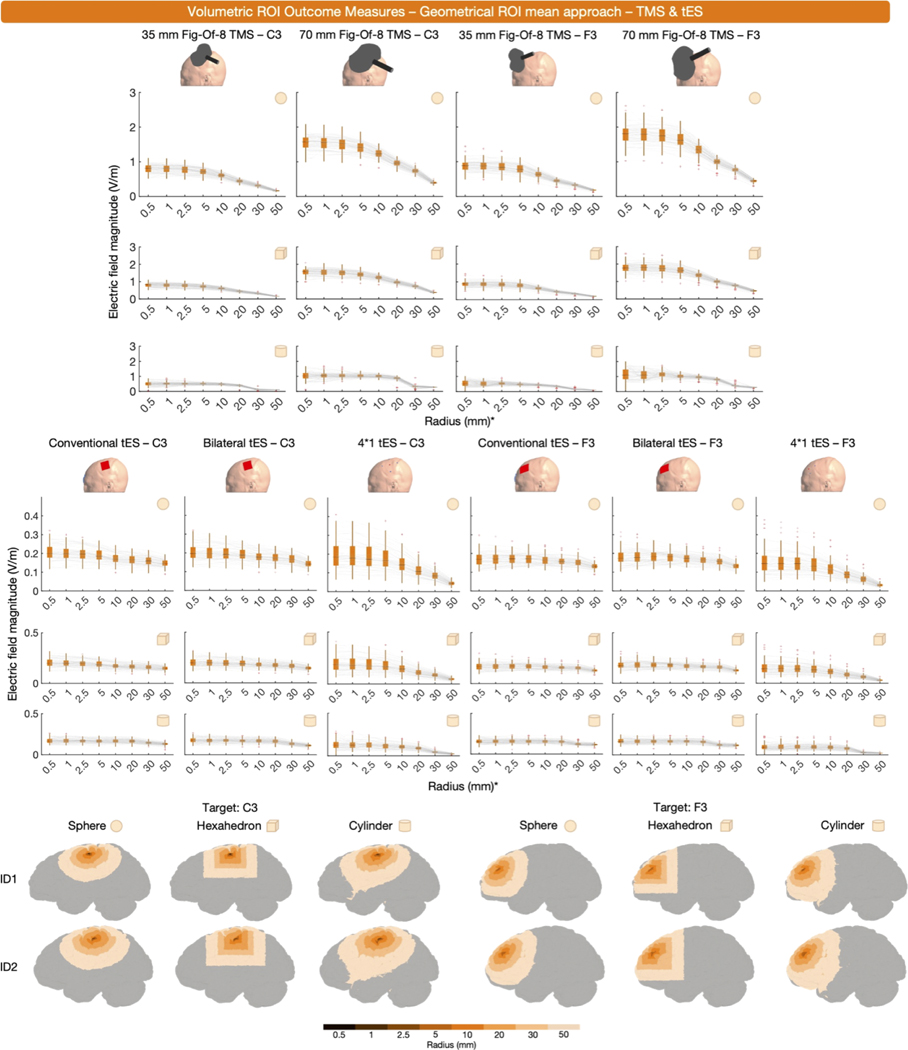
Volume-level, geometrical ROI, mean outcome measures obtained via spheres (rows 1 and 4), hexahedra (rows 2 and 5) and cylinders (rows 3 and 6), centered over the cortical projections of C3 or F3. The boxplots demonstrate the impact of ROI size on the mean E-field magnitude. Rows 1 to 3 relate to TMS, while rows 4 to 6 relate to tES. Generally, larger ROIs result in smaller E-field magnitudes with cylindrical ROIs retrieving the lowest magnitudes due to their large size. The last two rows show geometric ROIs in 2 representative participants. *Concerning the hexahedral ROIs, the x-axes present the radii values of spheres with the same volume of the hexahedral ROI. This facilitates the comparison between spherical and hexahedral ROIs.

The results, shown in Fig. 5, reveal several characteristics of geometrical ROI analyses. First, it is evident that larger ROIs generally extract smaller mean E-field magnitudes. In addition, there is a modality-specific effect of ROI size. In non-focal montages such as conventional and bilateral tES, E-field magnitudes only substantially decrease when using ROIs with radii ≥ 30 mm since a broader amount of volume is stimulated at higher intensities. In focal montages, such as TMS and 4*1 tES, the effect of ROI size on E-field magnitude is more pronounced, as ROIs with radii exceeding 10 mm already result in strongly attenuated E-fields due to extracting regions outside of the stimulation focality.

Fig. 5 indicates that spherical and hexahedral ROIs retrieve similar E- field magnitudes, implying that the choice between the two should be based on the experimental paradigm. Spherical ROIs have the advantage of equal distance to the center coordinate in all directions, whereas hexahedral ROIs offer an intuitive link to voxel-based neuroimaging. In contrast, cylindrical ROIs consistently result in lower E-field magnitudes (Fig. 5), due to the larger and deeper volumes encapsulated in these ROIs. For example, a 10 mm cylindrical ROI over C3 had a mean size of 3.45 ± 0.33 cm^3^, whereas the average size of the 10 mm spherical and hexahedral ROIs over C3 were 0.17 ± 0.03 and 0.17 ± 0.04 cm^3^, respectively.

In sum, geometrical ROIs are commonly used, and have the advantages of user-defined spatial specificity and a relatively similar volume extracted between individuals and studies. However, the risk of geometrical ROIs is that their utility depends on the researcher selecting a relevant brain region. If the ROI is restricted in size, does not capture the region of interest and/or relevant E-fields, the information yielded by the ROI may be less informative particularly for more diffuse, less focal stimulation montages. Likewise, if a large ROI is selected for a focal stimulation montage such as 4*1 tES, the E-field magnitude can drop off quickly due to non-stimulated regions being encapsulated within the ROI (Fig. 5). Thus, while geometrical ROIs can be highly useful and are easily implemented, their drawbacks must be considered. As with structural ROIs, there may be utility in combining ROI-based and percentile outcome measures to capture both the E-field magnitude at the cortical target and the whole brain maximal E-field values.

#### 3.2.3. Direct comparison of volume-level outcome measures

The abundancy of methods to quantify E-fields at the volume-level raises the question if E-field magnitudes obtained through different outcome measures are related. If so, this could imply that the choice between certain outcome measures is somewhat less important, although highly correlated values may still vary in absolute terms. If different outcome measures do not correlate, this would highlight that the selection of a particular outcome measure should be carefully considered as they highlight considerably different E-field features. This becomes particularly relevant as E-field modeling is increasingly used to determine dose-response relationships, identify which brain regions and/or persons receive the largest E-field magnitudes, and elucidate which stimulation montages induce the highest E-field magnitudes.

To assess the correlation between different E-field outcome measures, we conducted Pearson correlation analyses. We examined the correlations between the following measures: whole brain percentile, whole brain mean, ROI structure mean, ROI structure percentile, ROI spherical mean, ROI hexahedral mean and ROI cylindrical mean (cf., previous results). For the geometrical ROIs, we used the 10 mm radius sphere, its volume matching hexahedron and the 10 mm radius cylinder, and the sphere volume matching hexahedron. The 10 mm radius-related geometrical ROIs were selected as these are most widely utilized in literature. For the percentile approaches, we used the 99.999th percentile as this was the best representation of the peak E-field (i.e., highest percentile) that showed lower signs of erroneous values (cf., Figs. 2 and 3).

**Fig. 6.**
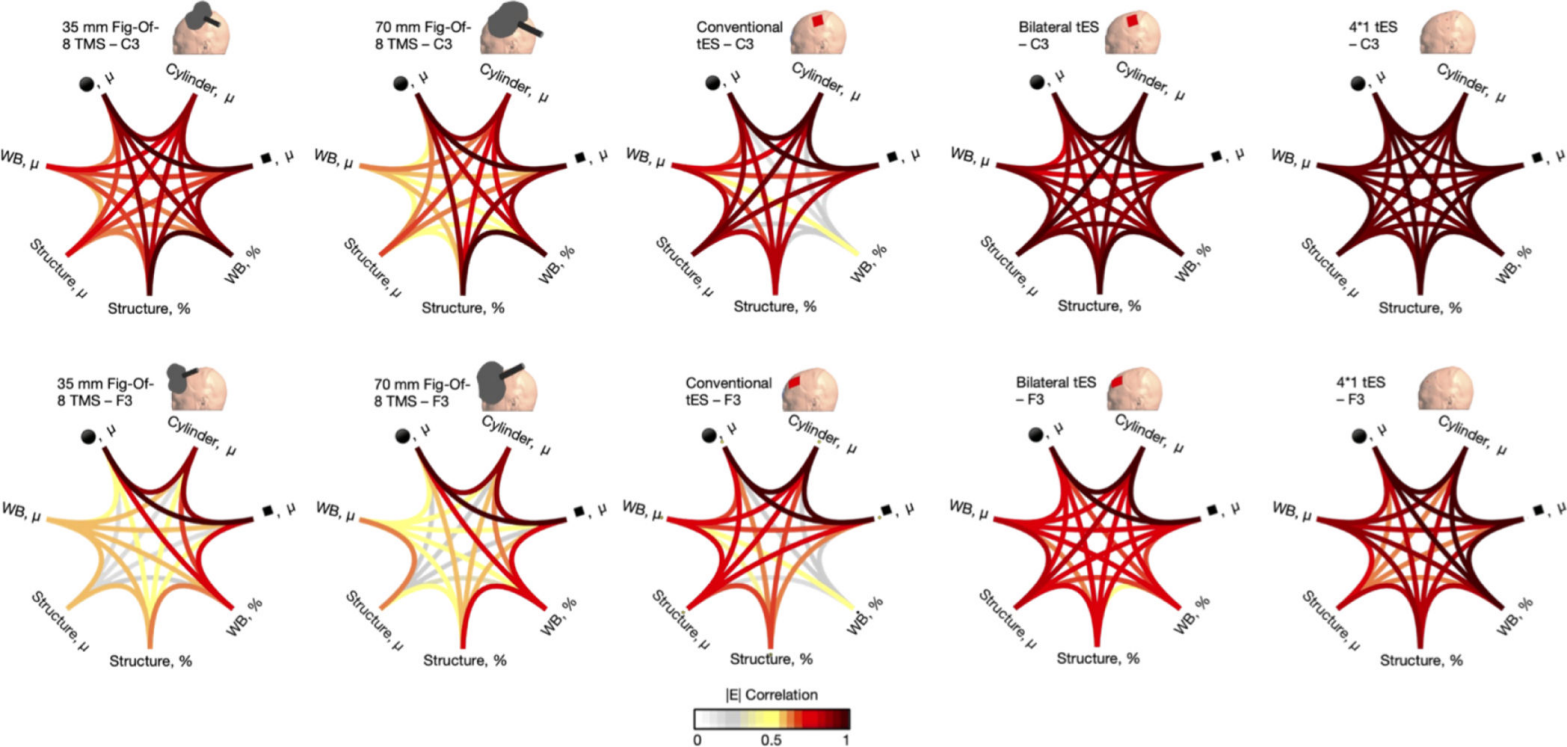
Pearson r correlation between different volume-level outcome measures. Unique correlation patterns are observed for each modality and target region (C3 [upper row] or F3 [lower row]). The percentile approach (%) used the 99.999th percentile. The cylindrical and spherical ROIs used 10 mm radii, while the hexahedral ROI matched the volume of the spherical ROIs. μ = mean, WB = whole brain, ■ = hexahedron, ●= sphere.

Fig. 6 shows modality- and target- specific Pearson correlation matrices. The overall correlation (mean ± standard deviation) was *r* = 0.74 ± 0.21. However, the lowest and highest correlations were *r* = 0.08 and *r* = 1.00, demonstrating a large range of values and underscoring the importance of well-selected outcome measures. When exclusively considering the correlations including the structural ROI outcome measures, 89.09 % of the correlations were higher when targeting F3 compared to C3. This highlights that the regional specificity of structural ROIs, as the C3 structural ROI correlated more to the other C3 outcome measures (i.e., the precentral gyrus) compared to F3 (i.e., middle frontal gyrus) (Cf., Section 3.2.2.1).

The strongest correlations were consistently present between the spherical mean and hexahedral mean approaches, which aligns with the results presented in Section 3.2.2.2.. Across all ten E-field models, the geometrical ROIs correlated strongly, with Pearson’s correlations ranging between *r* = 0.83 and *r* = 1. Within the group of geometrical ROIs, the weakest correlations were present between the sphere/hexahedron and the cylinder, yet even these outcomes were highly correlated. As E-field values retained from the cylindrical ROIs were considerably lower than those retained from the spheres and hexahedra yet had strong correlation values (Fig. 5), this demonstrates that E-field values do not need to be identical in absolute terms, to still capture the same overall E-field dynamics.

For all TMS modalities, geometrical ROIs, and/or percentile whole brain outcome measures exhibited the strongest correlations with each other (Fig. 6). The whole brain and structural mean outcome measures on the other hand, correlated considerably less well with all other outcome measures compared to all the other between-outcome measure correlations, indicating that these should best not be used for focal TMS. Moreover, the 99.999th percentile structural ROI outcome measure for TMS over F3 also had a relatively weak correlation to the other F3 outcome measures, implying that the structural ROI selection for F3 may have been less optimal compared to ROI selection for C3 montages (mean *r* = 0.76 ± 0.18).

For conventional tES over C3 and F3, the whole brain 99.999th percentile outcome measure correlated poorly to all other outcome measures, with a mean correlation across all modalities and outcome measures of Pearson’s *r* = 0.32 ± 0.09. These findings complement prior literature demonstrating that the maximal E-field for conventional tES approaches is not directly underneath the anodal electrode (D Antonenko et al., 2021; S Van Hoornweder et al., 2022; Ghazaleh et al., 2022), and further demonstrate that for non-focal modalities, the whole brain percentile approach provides fundamentally different information than all other outcome measures. For both bilateral and 4*1 tES, the volumetric outcome measures strongly correlated with each other, overall (mean *r* = 0.87 ± 0.10).

### 3.3. Surface-level outcome measures

In contrast to volume-based approaches, other studies have used surface-level outcome measures as is covered in Section 3.3. On the surface-level, E-field magnitude was extracted 111 times, across 54 studies. While surface-level data does not reflect the volumetric nature of the brain, it allows researchers to define the direction of the E-field with respect to the cortical surface, which may be informative as E-field direction is known to influence the effects of TMS and tES. A substantial number of surface-level outcome measures quantified normal (*n* = 34) or tangential (*n* = 13) E-fields, with the remaining outcome measures (*n* = 64) quantifying the net E-field magnitude (averaging across individual components). While the surface-level can be defined in several ways, we used the middle gray matter surface in line with the standard SimNIBS approach). An overview of the outcome measures used is shown in Fig. 1 and Table 3.

#### 3.3.1. Surface-level, whole brain outcome measures

Surface-level whole brain E-fields were quantified in 42 instances (Fig. 1). The majority of these measures were related to tES (*n* = 36) with a smaller portion related to TMS (*n* = 6). Overall E-field magnitude was extracted 25 times whereas the normal and/or tangential components were extracted 17 times.

Percentile-based extraction was used 32 times, as measured by the 75th, 90th, 95th, 97th, 98th, 98.5th, 99th, 99.9th, 99.99th or 100th percentile. Similar to the volumetric whole brain analyses, some outcome measures (*n* = 24 across 16 studies) used the 100th percentile in isolation or combination, or did not specify the specific percentile representing the peak (SM Rampersad et al., 2014; Jiang et al., 2020; Bortoletto et al., 2016; Csifcsák et al., 2018; Cvetković et al., 2015; Cvetkovic et al., 2023; Kessler et al., 2013; Laakso et al., 2015; Metwally et al., 2015; Miranda et al., 2013; N Mittal et al., 2022; N Mittal et al., 2022; Splittgerber et al., 2020; Zmeykina et al., 2020; Im et al., 2019; Yuan et al., 2023). For surface-based whole brain E-field extraction, we obtained the 99th, 99.9th, 99.99th, 99.999th and 100th percentile values of E-field magnitude, and the inward and outward E-field normal and E-field tangential components from all participants and simulations.

**Fig. 7.**
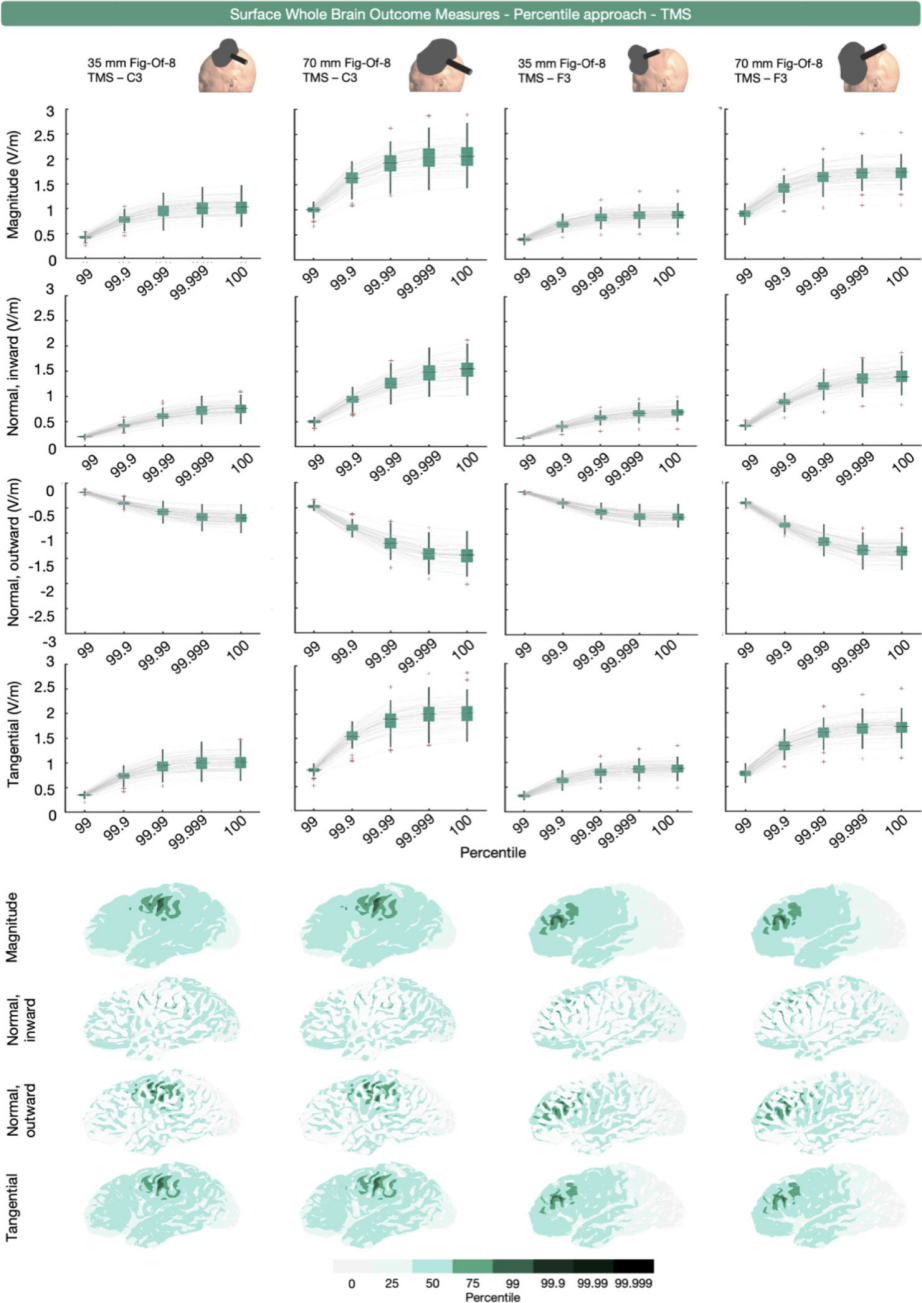
Surface-level (middle gray matter surface), whole brain, percentile-based outcome measure for E-field magnitude, and the normal and tangential components induced by TMS over C3 and F3 with 35 and 70 mm figure-of-eight coils. The upper four rows show the extracted E-field magnitude values using top percentiles. The lower four rows show the areas analyzed per percentile in a representative participant. Substantial variations in the extracted areas are observed across the different E-field components.

**Fig. 8.**
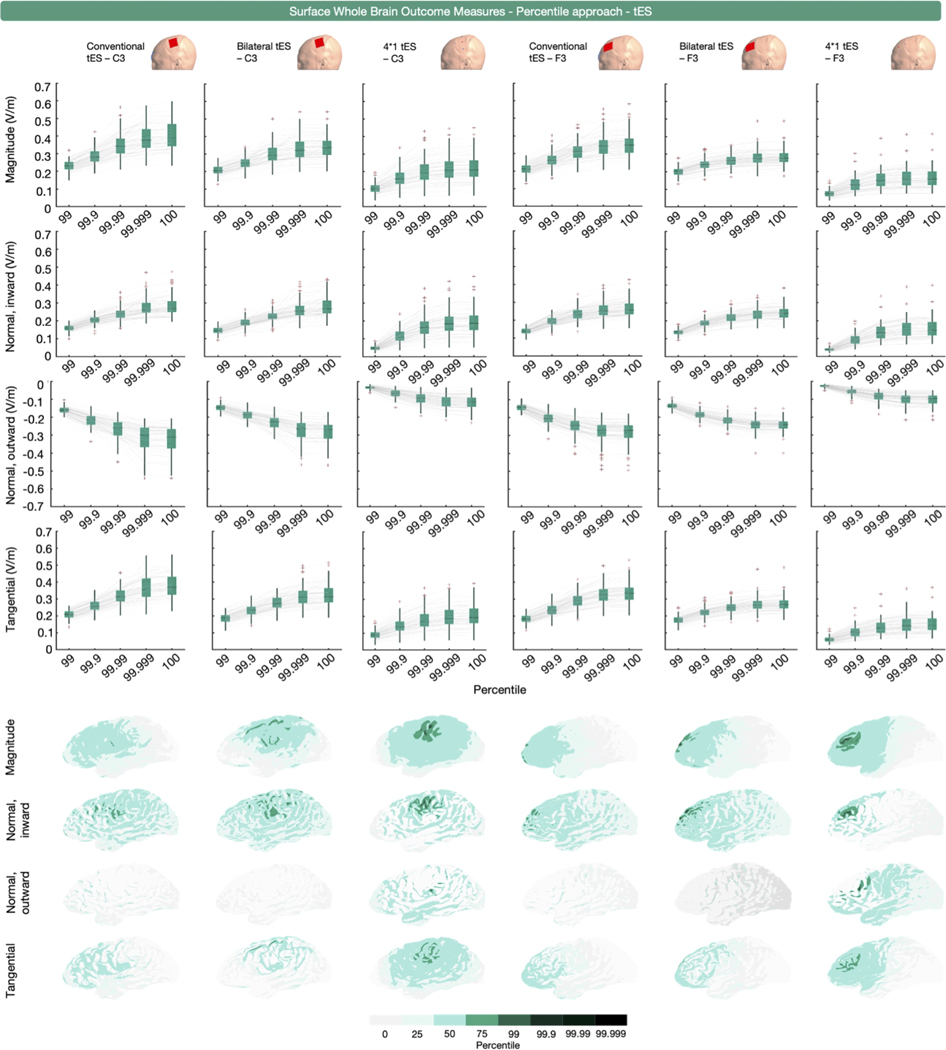
Surface-level (middle gray matter surface), whole brain, percentile-based outcome measure for E-field magnitude, and the normal and tangential components induced by conventional, bilateral, and 4 × 1 tES over C3 and F3. The upper four rows show the extracted E-field values using the top percentiles identified in our systematic review. The lower four rows show the areas analyzed per percentile in a representative participant. Substantial variations in the extracted areas are observed across the different E-field components.

Figs. 7 and 8 present the TMS and tES modeling results. Unlike the volume-level data, the surface-level data show no erroneous deviations at the 100th percentile. This is due to the surface being the middle-gray matter surface, while the volume-level includes the outer gray matter layer. As the outer gray matter surface interfaces with the cerebrospinal fluid, it therefore includes electric field values related to staircasing errors stemming from the difference in conductivities between both tissues (see (Gomez et al., 2020; Laakso and Hirata, 2012) for more information).

For TMS, all E-field components exhibited similar, coil-specific trajectories with the 70 mm figure-of-eight coil producing the highest absolute values across all E-field outcome measures. Moreover, E-fields resulting from TMS over C3 compared to TMS over F3 were larger across all components, for both TMS coil types, in absolute terms.

For tES, conventional tES applied over F3 and C3 resulted in the highest E-field magnitude, with the largest percentile value observed for conventional tES over C3. This was also true for the inward normal component, the outward normal component, and the tangential component, when considering absolute values. It is worth noting that the magnitude of the differences across tES modalities varied across different E-field components. For instance, the difference in 99.999th percentile values for bilateral and conventional tES over C3 were substantially larger for the E-field magnitude, the outward normal component, and the tangential component (mean differences of − 0.06, 0.05, and − 0.06 V/m, respectively), than for the inward normal component (mean difference = − 0.01 V/m).

The area covered by the percentile-based surface whole brain approach varies across persons, target region, modality and E-field component (Figs. 7 and 8). Similar to the volumetric results (Fig. 2), the inherent spatial agnosticism of this approach leads to spatial uncertainty about the analyzed region, particularly in non-focal montages such as conventional and bilateral tES for which the maximal E-field is not immediately underneath the anodal electrode. When analyzing multiple E-field components, an extra level of spatial uncertainty arises, as the same percentile corresponds to different brain regions depending on the selected E-field component (Fig. 8).

In sum, the surface-level whole brain percentile approach enables the examination of specific components of the E-field magnitude relative to the cortical surface, including normal and tangential components. The benefits and drawbacks of the surface-level whole brain percentile approach are similar to those in the volume-level whole brain percentile approach, including spatial agnosticism and how that can inform the overall cortical intensity but report off-target effects at certain percentile thresholds in more diffuse types of stimulation.

**Fig. 9.**
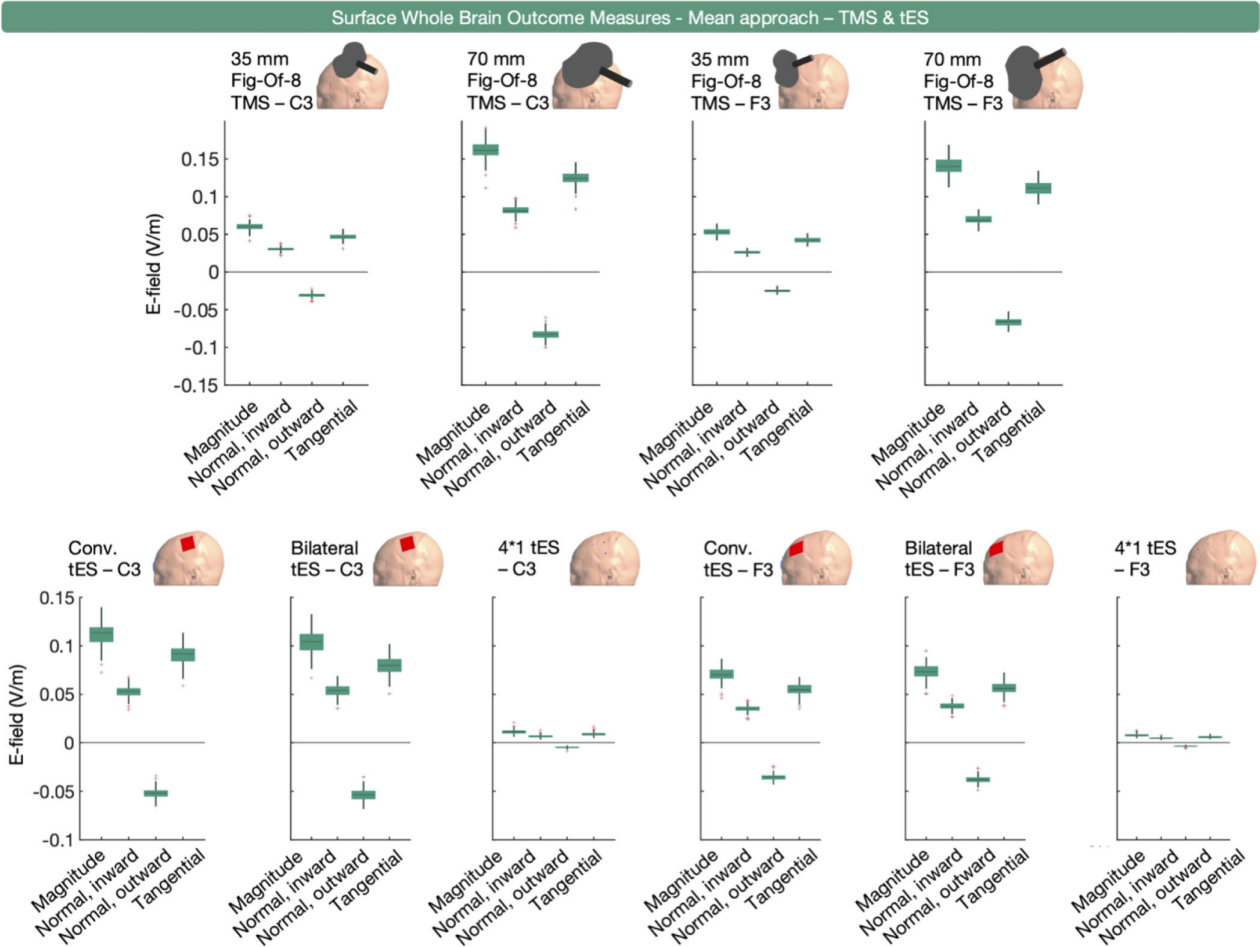
Surface-level (middle gray matter surface), whole brain, mean E-field magnitude and normal inward, normal outward, and tangential E-fields for ten TMS and tES montages targeting C3 or F3. Across the different modalities, the overall E-fields induced when the montage is set over C3 are larger compared to those in F3, in absolute terms.

Mean surface-level data was extracted in 10 TMS coil and tES electrode montages, and is shown in Fig. 9. Consistent with its volume-level counterpart, the informativeness of the surface-level mean outcome measure is limited to reporting on the general exposure to an E-field. For element-wise E-field extraction, which was used once, we refer to 3.2.1. Volumetric whole brain analyses, as to why this approach was not modelled.

#### 3.3.2. Surface-level, region of interest outcome measures

A total of 69 outcome measures used a surface ROI approach to extract E-field data (Fig. 1). Among these measures, 17 were related to TMS and 52 to tES.

##### 3.3.2.1. Surface-level, structural region of interest outcome measures.

**Fig. 10.**
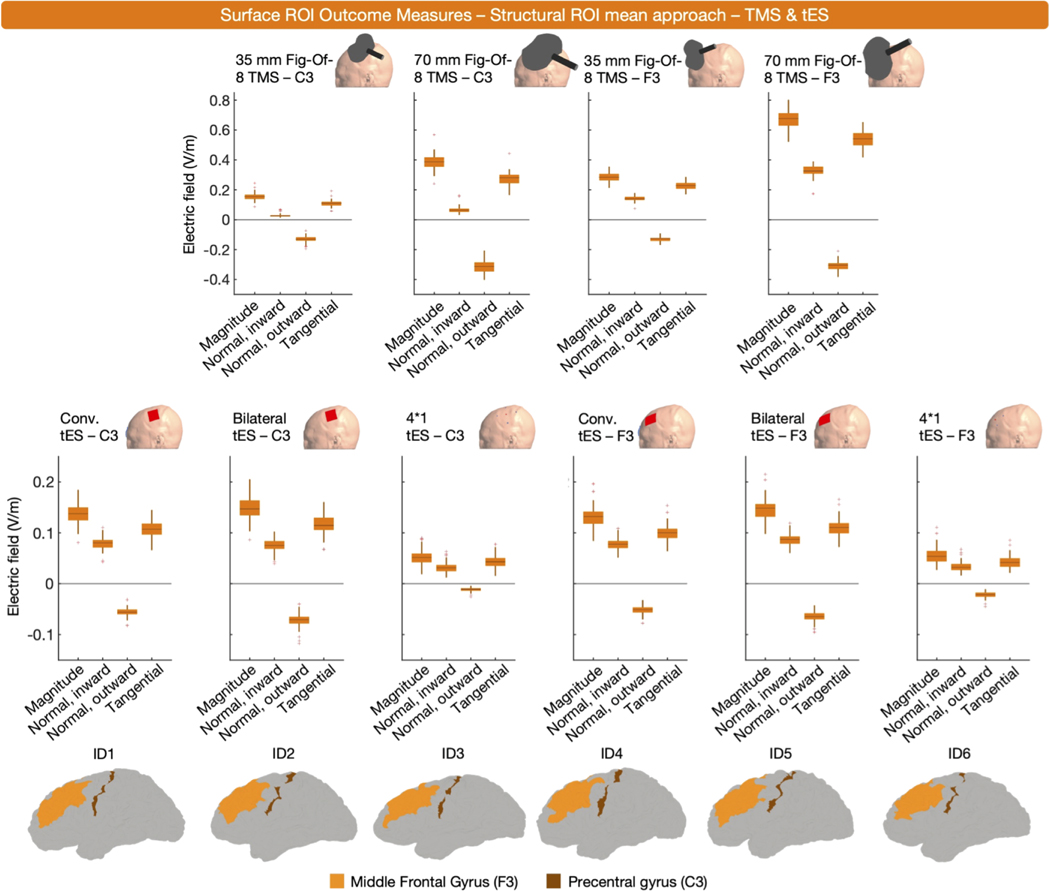
Surface-level (middle gray matter surface), structural ROI, mean E-field magnitude and normal inward, normal outward and tangential E-fields for ten TMS and tES montages targeting C3 or F3. The lowest row shows the used structures in 6 representative participants. Similar to its volume-level counterpart, the E-fields induced by bilateral tES are stronger in magnitude than those induced conventional tES. Also, both TMS coils targeting F3 induce stronger E-fields compared to their counterpart targeting C3.

**Fig. 11.**
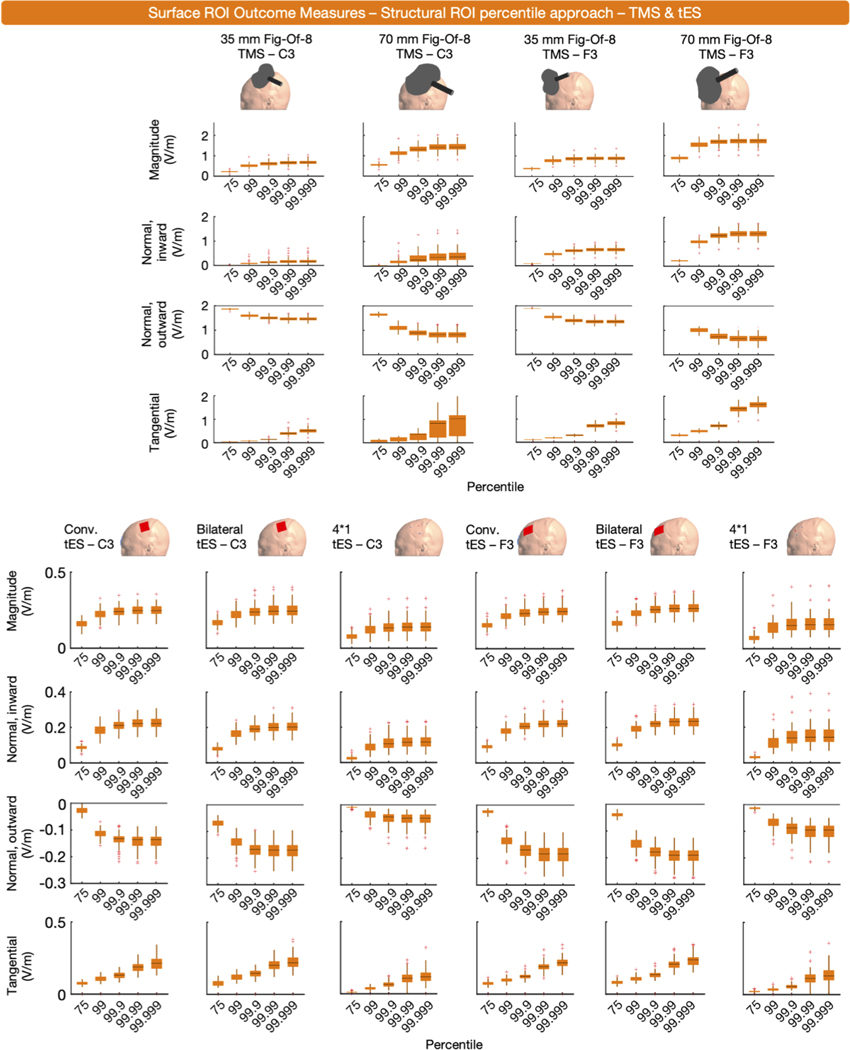
Surface-level (middle gray matter surface), structural ROI, percentile E-field magnitude and normal inward, normal outward and tangential E-fields for ten TMS and tES montages targeting C3 or F3. For each component and modality, the effect of different percentiles is shown.

The majority of ROI outcome measures used a structural ROI (*n* = 47). Among these, E-fields were extracted either as mean (*n* = 22) or percentile values (*n* = 25). Concerning the latter, the 0th, 50th, 60th, 80th, 90th, 98th, 99.9th, 99.999th, and 100th percentile were used. To inform on surface-level structural ROIs, we established subject-specific structural ROIs via the human connectome project (HCP) Multi-Modal Parcellation atlas and the SimNIBS ‘subject-atlas’ function. The left DLPFC (regions 46, 8Ad, 8Av, 8BL, 8C, 9–46d, 9a, 9p, a9–46v, i6–8, p9–46v, s6–8 and SFL) and M1 (region 4) were used as ROIs for tES and TMS targeting F3 and C3, respectively (Cf., Fig. 10) (Huang et al., 2022). From these ROIs, we extracted the mean E-field values and 99th, 99.9th, 99.99th and 99.999th percentiles of all four E-field components. Fig. 10 shows the results of the mean approach, Fig. 11 shows the results of the percentile approach.

Despite using different structural ROIs, some findings remain consistent across the volume and surface-level structural ROI data. For instance, in both ROIs, the mean E-field magnitude induced by TMS is stronger in F3 compared to C3. Also, both ROI analyses indicate that bilateral tES generates higher E-field magnitudes in the ROI, compared to conventional tES. However, discrepancies between the structural volume and surface ROIs are also prevalent. In the volume-level analysis (Fig. 4), E-field magnitudes are notably higher for tES targeting C3, compared to F3. Conversely, in the surface-level analysis, this difference is minimal-to-opposite. This discrepancy may be caused by the use of different atlases, highlighting that structural ROIs based on different atlases can lead to different conclusions, depending on their composition (e.g., the volume-level data suggests that TMS over C3 results in higher E-field magnitudes, whereas the surface-level data suggest that TMS over F3 results in higher E-field magnitudes). Although other factors such as a generally thicker gray matter layer in the F3 region (i.e., a deeper middle gray matter layer) may also play a role (Van Hoornweder et al., 2023).

To summarize, surface-level structural ROIs were used to extract the mean or percentile value E-fields and have similar strengths and drawbacks as volume-level structural ROIs. The use of surface-level structural ROIs for E-field modeling is highly dependent on the researcher’s selection of a relevant brain target. As with other outcome measures, it is important to consider how the size of the structural ROI may impact E- field magnitude, both overall and in specific components.

##### 3.3.2.2. Surface-level, geometrical region of interest outcome measures.

Spherical ROIs were used to extract surface-level E-field data 15 times (6 TMS and 9 tES outcome measures). E-field magnitude was extracted 5 times, and the normal and/or tangential E-field components were extracted 10 times. The E-field within the sphere was quantified as the mean in 13 instances, as the mode once, and as a percentile once (i.e., the peak E-field). The radius of the used sphere varied, with radii of 2 mm, 3 mm, 5 mm, 10 mm and 5 elements being used.

Hexahedral ROIs were used 7 times, across two studies. Either the mean (*n* = 4) or the 95th percentile (*n* = 3) was extracted. The size of the hexahedron was 1*1*1 mm^3^ or 2*2*2 mm^3^.

**Fig. 12.**
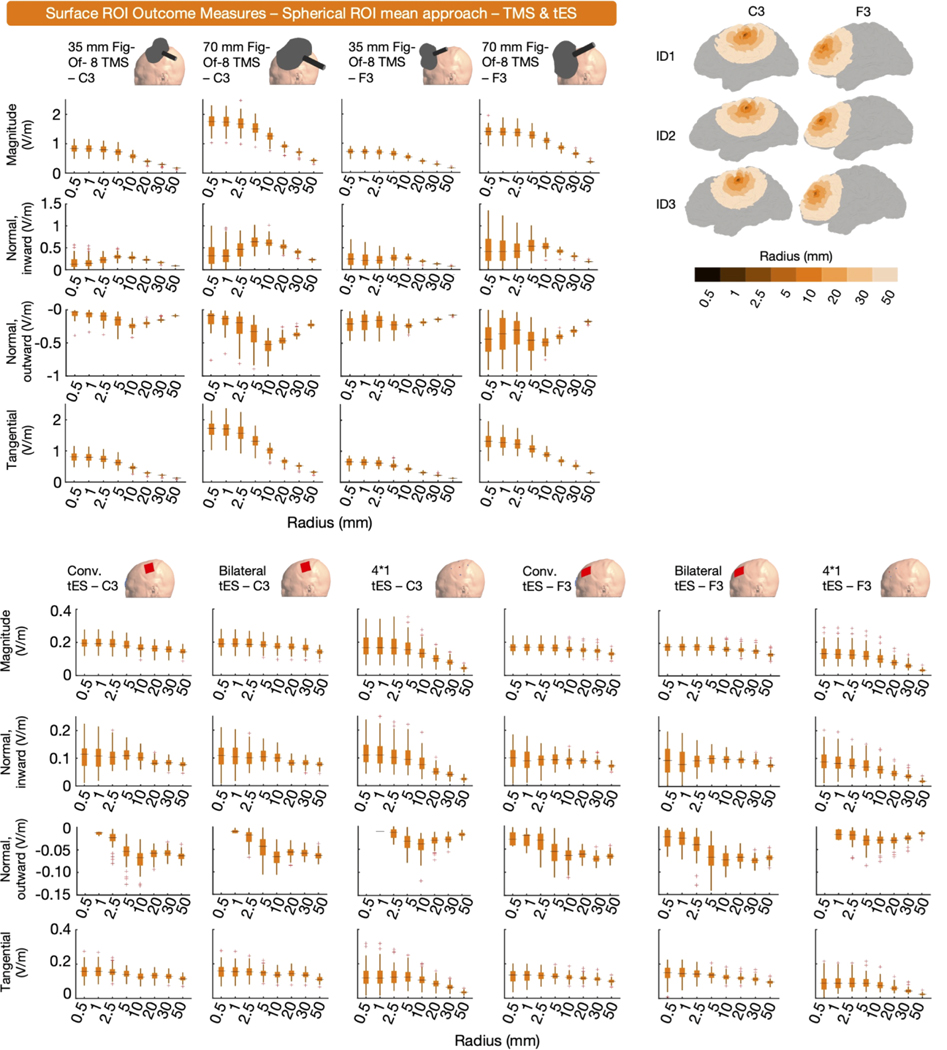
Surface-level, spherical ROI, mean E-field magnitude and normal inward, normal outward, and tangential E-fields for ten TMS and tES montages targeting C3 or F3. The effect of different sphere sizes is shown per modality and E-field component. Particularly for the normal inward and outward components, the retrieved results differ from the volume-level data in that in some instances, larger ROI sizes results in greater absolute E-field values.

To inform on the utility of geometrical surface-level ROIs, we extracted the mean of the four E-field components via spherical and hexahedral ROIs from all participants and montages. For montages targeting C3, we centered the ROIs over the middle gray surface node closest to the subject-space transformed cortical projection of C3 (MNI coordinate = − 52.2, − 16.4, 57.8). For montages targeting F3, the subject-space transformed coordinate of the cortical projection of F3 (MNI coordinate = − 35.5, 49.4, 32.4) was used. All shapes only extracted E-fields on the middle gray matter surface. All ROIs of the same shape only differed in terms of size. Concerning the spherical ROIs, radii were 0.5, 1, 2.5, 5, 10, 20, 30, and 50 mm. The side length of the eight hexahedral ROIs was configured so that each hexahedral ROI was a cube matching the volume of spherical ROIs if both ROIs were entirely filled with gray matter. The hexahedral ROIs were positioned along the X, Y and Z axes. The results of the spherical ROIs are shown in Fig. 12. We did not plot the results of the hexahedral ROIs as these resulted in nearly identical values as the spherical ROIs, similar to the volumetric hexahedral ROIs and corroborated by the analyses in Section 3.2.3.

Concerning E-field magnitude and the tangential E-field, the results mainly seem to corroborate the volume-level geometrical data in that larger shapes average out E-field data, and the effect of ROI size depends on the focality of the montage. The normal inward and outward E-field components exhibit different characteristics. Specifically, for the normal outward component in both TMS and tES, as well as the normal inward component in TMS, the highest absolute peak values were observed with ROI sizes ranging from 5 to 10 mm. This suggests that smaller ROIs (< 5 mm) did not capture the peak normal E-field in certain TMS and tES modalities, which is reasonable as the purpose of ROI analyses is not to specifically capture the peak fields but rather to calculate the field in an ROI, regardless of whether it includes the peak or not.

In summary, similar to volume-level analyses, researchers have used geometrical ROIs in surface space. This analysis enables the examination of E-field components relative to the cortical surface which is not possible in volume space. As is true in the volume space analyses, ROIs are beneficial when researchers seek to analyze the E-field intensity within a specific targeted region but have the potential drawback of possibly missing overall maximal E-field values that may be better captured by whole brain percentile analyses. As aforementioned, missing the maximal E-field values is not necessarily problematic, but rather depends on the hypotheses and aims of the researcher. Furthermore, geometrical ROIs are not individualized in the sense that they do not take anatomy and/or functional data into consideration, which can be achieved through structural ROIs.

#### 3.3.3. Direct comparison of surface-level outcome measures

To assess the consistency of surface-based outcome measures in retrieving similar E-field values, we calculated Pearson’s r correlations for each E-field component between the whole brain percentile, whole brain mean, ROI structure mean, ROI structure percentile, ROI spherical mean, and ROI hexahedral mean outcome measures. For the geometrical ROIs, a 10 mm radius sphere and its volume-matching hexahedral counterpart were used. For the percentiles, the 99.999th percentile was used consistent with the volume analyses (Fig. 2).

**Fig. 13.**
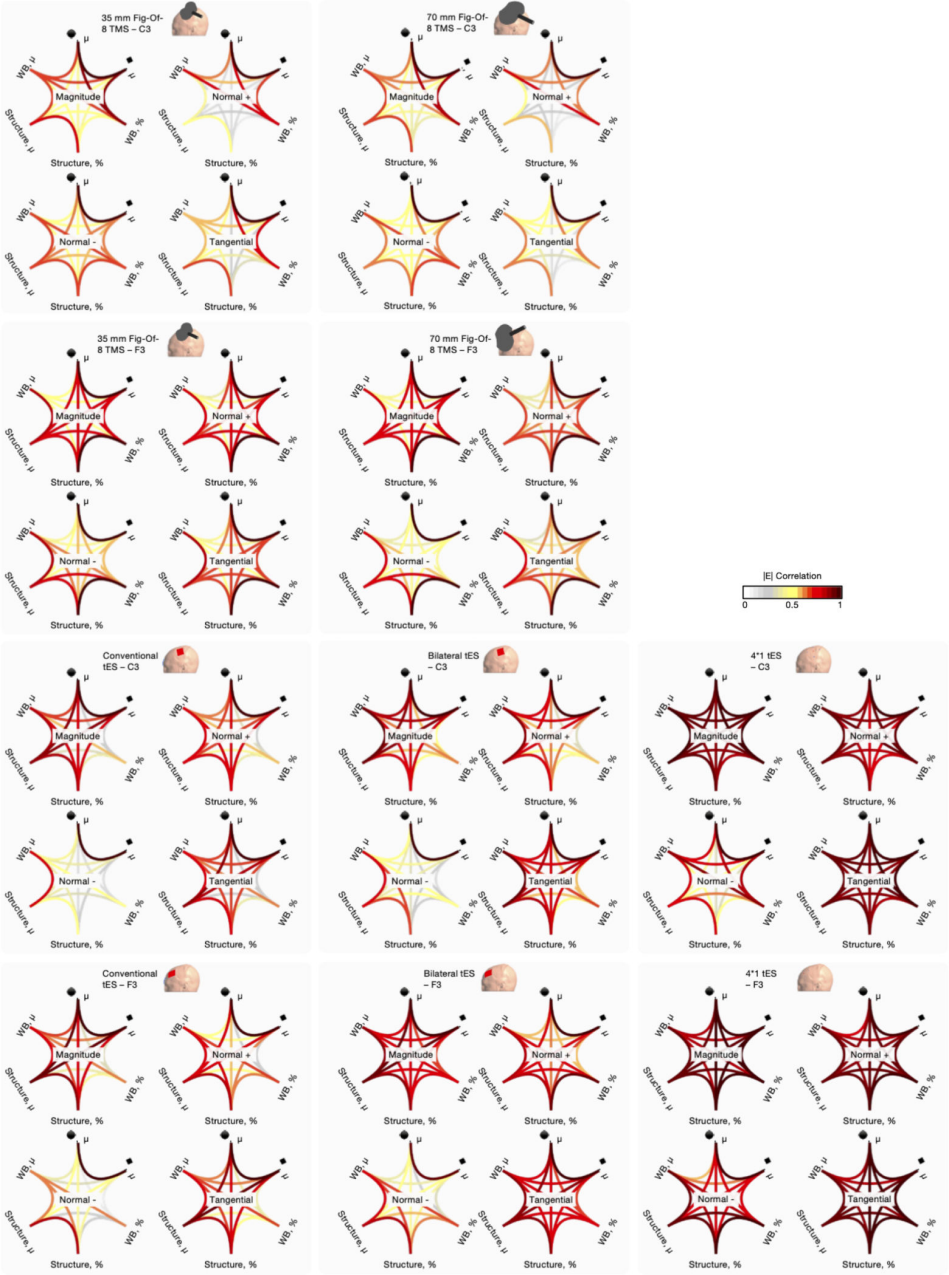
Pearson correlations showing how the different surface outcome measures relate. Overall, unique correlation patterns were found per modality, targeted region and E-field component. For the percentile approach (%), the 99.999th percentile was used. For the spherical ROIs, 10 mm radii were used. The hexahedral ROI was established to match the volume of the spherical ROI. Normal + and – = inward and outward normal E-field, respectively, Struct. = structure, μ = mean, WB = whole brain, ■ = hexahedron, ● = sphere.

Similar to the volume-level analyses, there was a wide range of correlations between outcome measures from *r* = 0.02 to 1.00 (mean *r* = 0.67). The correlation patterns shown in Fig. 13 are unique and dependent on the targeted region, modality, and E-field component. These data directly demonstrate the importance of considering outcome measure when comparing E-field modeling results across studies. Overall, between outcome measure correlations were highest for 4*1 tES (mean Pearson’s r value across all modalities and E-field components = 0.88 ± 0.06), corroborating the volume-level data that for focal tES, outcome measure selection may be less critical compared to other modalities, at least using the 99.999th percentile compared to ROI approaches. Across the board, the weakest between outcome measure correlations were present for TMS with the 70 mm figure-of-eight TMS (mean Pearson’s r value = 0.61 ± 0.17). Also similar to the volume-level data, the spherical and hexahedral mean outcome measures were strongly correlated (global mean Pearson’s r value = 0.99 ± 0.01). The weakest correlation was present for conventional tES targeting C3, where the normal outward component value retrieved by the whole brain percentile approach and structure ROI percentile approach had a Pearson correlation value of *r* = 0.02.

Across all modalities, the lowest correlations between outcome measures were retained for the outward normal component (Pearson’s *r* = 0.58 ± 0.14). The inward normal component (Pearson’s *r* = 0.66 ± 0.10), E-field magnitude (Pearson’s *r* = 0.74 ± 0.09) and tangential component (Pearson’s *r* = 0.70 ± 0.10) correlated better across the board. The difference between the normal inward and outward components may be attributed to the tES montages having the anodal electrode over scalp locations C3 / F3, thereby inducing a predominant normal inward component in the ROIs.

In sum, these correlations directly demonstrate the importance of selecting outcome measures that are well-suited to addressing the research question of interest. Even within the same participant and same model, the selected outcome measure can substantially alter the interpretation of data with an overall average Pearson’s correlation value of *r* = 0.67 and wide range of *r* = 0.02 to 1.00. With our systematic review finding that there is marked variation in E-field outcome measures used, it is important to consider how the field can report more consistent and comparable results across studies. The focus on improving reporting standards and summarizing the pros and cons of different E-field measures is the topic of Section 4.1.

## 4. Discussion

In this combined systematic review and large-scale E-field modeling study, we analyzed how the selected outcome measure impacts E-field quantification on the volume and surface-level. In the studies reporting 308 E-field outcome measures that fit our systematic review criteria, outcome measures fell into two major categories: ROI and whole brain approaches (Fig. 1). To substantiate the differences in outcome measures, we computed over 1000,000 E-field related outcome measures in 100 participants, demonstrating that the selected outcome measure significantly impacts the result of E-field quantification on the same E- field models. More specifically, in both volume- and surface-level analyses, the Pearson coefficient values in the same participants and same E- field models widely varied between 0.08 and 1.0 across outcome measures. Since there is as low as an 0.08 correlation coefficient between different outcome measures on the same model, we highlight the need to rigorously consider how the selection of E-field outcome measure affects the interpretation of modeling data. An in-depth quantitative and qualitative exploration of each outcome measure was achieved in the results section. Here, we provide a brief overview of our findings.

When selecting outcome measures, the first choice that should be made is whether to analyze the data on the volume- or surface-level (cf., Results, Sections 3.2. and 3.3.). Whereas the volume-level has the advantage of better representing the gray matter volume, the surface- level can inform the direction of the E-field, albeit that directionality can also be investigated on the volume-level via interpolation (SM Rampersad et al., 2014) or tractography (Opitz et al., 2011). Surface-level analyses performed on surfaces that are not adjacent to other tissues such as the cerebrospinal fluid also bear the advantage that erroneous E-field values present due to staircasing errors are absent (cf., 3.3.1. Surface-level, Whole Brain Outcome Measures). Notably, this advantage is not an inherent characteristic of surface-level analyses, as it is mitigated when the selected surface neighbors other tissues.

Second, E-fields can be quantified on the whole brain level or by means of an ROI. We showed that whole brain analyses are most informative for focal stimulation methods when using high percentiles since this focuses the analysis underneath the center of the electrode or coil. Notably however, the 100th percentile, a commonly utilized approach in the literature, is less accurate when the gray matter – cerebrospinal fluid boundary is incorporated in the volumetric model (Fig. 2). Concerning ROIs, numerous options are available. Structural ROIs were consistently the most widely used ROI approach, both on the volume- and surface-level (Fig. 1). These structural ROIs offer the advantage that they can be tailored to specific hypotheses, atlases and/ or neuroimaging data. However, this comes at the drawback that comparing various structural ROIs of the same region can be impeded by factors such as different shapes and sizes that can differ depending on individual anatomy. This drawback is attenuated by geometrical ROIs, which were most-often spheres (Fig. 1). Concerning these geometric ROIs (Figs. 5 and 12), which are less easily integrated with neuroimaging data, ROI size yields a modality specific impact, with more focal modalities being most affected by ROI size. Overall, spheres with radii up to 5 to 10 mm, depending on the modality, remained stable in terms of obtained E-field magnitude, whereas larger radii obtained increasingly lower E-field magnitudes. Concerning the other E-field components, the effect of geometric ROI size depended on the E-field component (Fig. 12). Finally, in both the volume and surface-level analyses, a recurring theme was that different outcome measures correlated to greater or lesser extents based on the focality of stimulation, the stimulation target, and the E-field component. For instance, Pearson correlation values on the volume-level for different outcome measures on the same E-field models ranged from only *r* = 0.08 to 1.0. Thus, the selection of outcome measure and its suitability to address the question of interest can directly affect the interpretation of the same E-field models. To begin to standardize the types of data reported in E-field modeling studies, we present four recommendations for more consistent E-field outcome reporting measures to improve the comparability between studies.

### 4.1. Recommendations for future research

It is clear that the selected outcome measures substantially affects the obtained E-field value and the interpretation of simulation results. Although ultimately, an outcome measure is always chosen in the context of specific research goals, there are some recommendations that can be made based on our review and data analyses that should be considered when deciding which outcome measure to use, and how to interpret E-field modeling study results.

#### Recommendation #1: The best practice is to use a combination of outcome measures and to visualize which regions each outcome measure analyzes.

One of the primary conclusions of the current work is that different outcome measures inform on different aspects of the complex vectorial fields comprising E-fields. The specific information provided by each outcome measure depends on various factors, such as TMS and/or tES modality, the individual, the target region, and the specific E-field component being studied. This is evident from the complex correlation patterns observed for both the volume and surface-level data (Figs. 6 and 13). Based on these findings, we strongly recommend that future studies incorporate multiple outcome measures, encompassing volume- and surface-space, as well as whole brain percentile and ROI approaches, to assess the robustness of their findings. By examining the results across different outcome measures, researchers can determine if their findings are consistent or if they are specific to a particular outcome measure. If a specific outcome measure is needed to detect specific results, the information about which E-field outcome measure explains the findings will be highly valuable to comprehend the origin and interpretation of the observed effects.

#### Recommendation #2: Use the Study Goal to Select the Most Suitable Outcome Measure

When selecting an outcome measure, it is key to consider the specific goal of the study and the available data. Different outcome measures have their strengths and limitations, and the choice for one should align with the research objectives. If neuroimaging data such as functional MRI is available, defining a volume-level, structural ROI based on these data may be a viable approach. On the other hand, if the study’s goal is to compare E-field normal components in different neural regions or across persons, a surface-level small spherical ROI may be best suited. As many studies currently only report one or a few outcome measures, as stated in Recommendation #1, it may improve between-study comparability if studies shift to using multiple outcome measures while considering their strengths, weaknesses, and suitability to address the study goals. While an all-encompassing overview of these strengths and weaknesses is unfeasible, due to the many factors that influence the behavior of an outcome measure, Table 4 offers a concise summary of the pros and cons associated with the three most-commonly used outcome measures.

#### Recommendation #3: The Dose-Response Relationship Between E-Field Magnitude and Clinical Outcome Must Only Be Compared Within a Singular Outcome Measure

A primary finding of our systematic review is that researchers have used many different approaches to quantify E-field magnitude. With the powerful tool of E-field modeling, a question that many of us seek to answer is whether there is a relationship between the induced E-field and clinical response. It is enticing to consider these results monolithically and pursue an elegant statement such as, “The optimal E-field is ___ V/m.” The simplicity of pursuing a singular value is appealing and would enable easier dissemination of individualized E-field dosing such as through dose-controlled tES, 2-Sample Prospective E-field Dosing (2- SPED), or applying an individualized TMS dose to induce a singular E- field at the cortical level across individuals (S Van Hoornweder et al., 2022; Evans et al., 2020). However, there are many reasons to believe that a singular optimal E-field value does not exist and that more nuance is necessary. For instance, a singular optimal E-field value would likely not apply in the same way across different brain regions due to varying neuronal composition, white matter tracts, and gray matter densities, among other variables (Glasser et al., 2016; McGrath et al., 2022; Josse et al., 2009; Cox et al., 2016). Age, sex, diagnosis, and other typical demographical considerations likely also impact the optimal E-field dose (D Antonenko et al., 2021; Indahlastari et al., 2020; S Van Hoornweder et al., 2021; Ghasemian-Shirvan et al., 2020; Ghasemian-Shirvan et al., 2022). Furthermore, the dynamic nature of the brain might further complicate things, as time-varying changes within a brain region of a single person may further modify the optimal E-field dose (Zatorre et al., 2012; Sampaio-Baptista et al., 2013). Here, our analyses suggest that outcome measure is a key consideration in any discussion of optimal E-field dose. The E-field extracted from the same models widely vary depending on the volume or surface area and regions considered. Thus, instead of the monolithic goal of a singular optimal E-field, we might instead work towards more nuanced goals taking many factors into consideration. In the future, we might come to the understanding that, “The optimal mean E-field for TMS, in a 5 mm radius spherical ROI on the volume-level, centered over the motor hotspot as defined by TMS, in 50- to 70-year-old adult patients with ataxia, is ___ V/m.” This value would almost certainly be different than an “optimal prefrontal E-field magnitude for TMS, measured by the 95th percentile whole brain approach on the surface-level, and in 20- to 40-year-old adult patients with depression.” Of course, additional refinement might further personalize our understanding of any “optimal” E-field value between individuals even when they have similar ages or diagnoses.

#### Recommendation #4: Time for Standardized E-Field Outcome Measure Reporting

Pursuing goals such as better understanding the relationship between E-field components and therapeutic outcome necessitates that researchers report more standardized outcome measures between studies. As highlighted in *Recommendation #3* and throughout this study, comparing some outcome measures in some modalities is akin to comparing apples to oranges. Thus, to work toward more suitable comparisons across studies, it is necessary to consider how to improve the consistency of reporting across studies. Notably, this recommendation does not conflict with *Recommendations #1 and #2* calling for researchers to apply multiple outcome measures and select those that suit the experimental question. Rather, these reporting standards can apply to specific experimental questions which require different modeling outcome measures.

While deriving a comprehensive list of standard reporting procedures is beyond of the scope of this study and warrants a consensus-based approach, we propose that future work adheres to the following reporting standards:

1. To state that a specific brain region was stimulated, researchers must include an ROI-based method and describe how the ROI was defined in the Methods section. We propose this guideline since the peak E- field intensity derived from the whole brain percentile approach does not always coincide with the intended stimulation target, particularly with less focal forms of brain stimulation.
2. When defining an ROI, we recommend that researchers report the MNI coordinate that the ROI is centered on to improve methodological reproducibility. In cases such as structural ROIs in which the researcher might individually define the ROI, an average MNI value should be provided when possible to aid in the reproducibility of findings and comparisons between studies.
3. Whether using an ROI or whole brain percentile approach, the volume of the analyzed tissue should be reported. This recommendation seeks to allow for some degree of comparison between ROI and whole brain percentile approaches since the reader should at least be able to determine whether a similar volume was analyzed (keeping in mind that even with similar volumes analyzed, the ROI and whole brain percentile approaches may analyze differing brain regions). Researchers should ideally also visualize which regions are analyzed by an outcome measure, with this being particularly important for percentile-based approaches to enable the reader to interpret the regions of the extracted volume.
4. Finally, we recommend all future studies to measure and report multiple outcome measures whenever possible. For instance, research applying conventional M1-SO tES could use both the spherical ROI approach if one is interested in a specific region, and the percentile-based approach with complementary visualizations, to assess whether peak E-fields were induced in the intended region. Moreover, as explicated in Section 3.2.1.1., the 100th percentile should not be used due to its incorporation of erroneous values that substantially deviate from nearby stepwise comparisons (e.g., 99.999th percentile)

### 4.2. Conclusions

Outcome measures in the computational noninvasive brain modeling field have received little attention in the past. Based on our systematic review and extraction of over 1000,000 E-field outcome measures, across 100 participants and 1000 tES and TMS E-field models, we show that different outcome measures substantially affect the obtained E-field magnitude and the analyzed brain region in a montage, person, and E- field component specific manner. Therefore, one should only interpret and compare E-field magnitudes across studies when similar outcome measure approaches are used. We formulated four recommendations and four reporting standards to ensure the informed selection of future outcome measures and informative reporting. Our hope is that adopting these recommendations and standards will help future work to avoid interpretational pitfalls and reduce the inconsistency of the used E-field outcome measures.

## Supplementary Material

1

## Figures and Tables

**Table 1:** Transcranial electrical stimulation (tES) and magnetic stimulation (TMS) montages.

	Bilateral tES	Conventional tES	4 × 1 tES	35 mm Fig-of-8 TMS	70 mm Fig-of-8 TMS

Target: C3	Anode: C3 Cathode: C4	Anode: C3, Cathode: FP2	Anode: C3, cathodes: C1, CP3, C5 & FC3	Coil over C3, pointing to FC1	
Target: F3	Anode: F3 Cathode: F4	Anode: F3, Cathode: FP2	Anode: F3, cathodes: F1, FC3, F5 & AF3	Coil over F3, pointing to FPz	
Intensity	Anode & cathode: 1 mA		Anode: 1 mA Cathodes: 0.25 mA	1 A/s	
Type	50 by 40 mm rectangular electrodes		5 mm diameter, circular electrodes	MagVenture MC-B35	MagVenture MC-B70

**Table 2:** Volumetric E-field outcome measures.

Component	Level	Type	Measure	N (TMS: tES)	Studies

|E|	Whole Brain		Percentile	70 (37:33)	(Turi et al., 2022; Wischnewski et al., 2021; SM Rampersad et al., 2014; Alekseichuk et al., 2019; Alam et al., 2016; Amani et al., 2021; Ashikhmin and Aliev, 2016; Carlson et al., 2023; Caulfield and George, 2022; Chung et al., 2022; Colella et al., 2021; Handiru et al., 2021; Hsu et al., 2023; Jiang et al., 2020; Kalloch et al., 2020; Kenville et al., 2020; Khorrampanah et al., 2020; Klaus and Schutter, 2018; Marquardt et al., 2021; McCann and Beltrachini, 2021; S Minjoli et al., 2017; Miranda et al., 2016; Padberg et al., 2021; Scheldrup et al., 2014; Seo and Jun, 2019; Shahid et al., 2014; Shahid et al., 2015; Soleimani et al., 2021; C Thomas et al., 2019; Uenishi et al., 2022; S Van Hoornweder et al., 2022; Wang et al., 2021; Woods et al., 2015; Wysokiński, 2021; Lu and Ueno, 2015; OF Afuwape et al., 2022; OF Afuwape et al., 2021; OF Afuwape et al., 2021; OF Afuwape et al., 2022; Alawi et al., 2023; Colella et al., 2019; S Fiocchi et al., 2016; S Fiocchi et al., 2016; J Gomez-Tames et al., 2019; Guadagnin et al., 2016; Guadagnin et al., 2014; Lee et al., 2016; Lewis et al., 2023; CS Li et al., 2019; Lu et al., 2023; Makarov et al., 2021; KE Mantell et al., 2021; McCalley and Hanlon, 2021; Rastogi et al., 2017; Rastogi et al., 2018; M Soldati and Laakso, 2020; F Syeda et al., 2017; F Syeda et al., 2017; F Syeda et al., 2017; M Tzirini et al., 2022; Tzirini et al., 2021; M Tzirini et al., 2022; Wang et al., 2018; Yamamoto et al., 2015; Zucca et al., 2017)
			Mean	11 (6:5)	(Huang et al., 2019; Amani et al., 2021; Ashikhmin and Aliev, 2016; Colella et al., 2021; Colella et al., 2019; KE Mantell et al., 2021; McCalley and Hanlon, 2021; Lang et al., 2020; Janssen et al., 2013; EG Lee et al., 2018)
			Element	7 (4:3)	(Kasten et al., 2019; Preisig and Hervais-Adelman, 2022; Steinmann et al., 2022; Germick et al., 2018; Zhong et al., 2022; Zhong et al., 2021; Zhong et al., 2019)
	ROI	Structure	Mean	30 (3:27)	(Suen et al., 2021; Lang et al., 2020; Steinmann et al., 2022; Bai et al., 2014; Benussi et al., 2021; Benussi et al., 2022; Cancelli et al., 2018; Esmaeilpour et al., 2020; Fernández-Corazza et al., 2020; Foerster et al., 2019; Indahlastari et al., 2018; Kalloch et al., 2022; Rauscher et al., 2020; Rezaee and Dutta, 2019; Rezaee and Dutta, 2020; Rezaee et al., 2019; Salameh et al., 2022; Salvador et al., 2015; Solanki et al., 2021; G Soleimani et al., 2022; Suzuki et al., 2022; C Thomas et al., 2019; Zanto et al., 2021; Zhang et al., 2021; Fischer et al., 2017; Salehinejad et al., 2020; Mezger et al., 2020; Mosayebi-Samani et al., 2021; Bhalerao et al., 2021; Gomez-Feria et al., 2022)
			Percentile	29 (9:20)	(Seo and Jun, 2019; Shahid et al., 2014; S Fiocchi et al., 2016; S Fiocchi et al., 2016; M Tzirini et al., 2022; Benussi et al., 2021; Benussi et al., 2022; Suzuki et al., 2022; C Thomas et al., 2019; Mezger et al., 2020; DaSilva et al., 2015; Datta et al., 2013; Edwards et al., 2013; Fiocchi et al., 2018; Fiocchi et al., 2022; S Fiocchi et al., 2016; WH Lee et al., 2018; Lu and Ueno, 2017; Manoli et al., 2017; Mizutani-Tiebel et al., 2022; Mosayebi-Samani et al., 2023; M Parazzini et al., 2017; Parazzini et al., 2015; Parazzini et al., 2014; Passera et al., 2022; Rezaee et al., 2020; Seibt et al., 2015; Thomas et al., 2018)
			Element	3 (3:0)	(Beynel et al., 2021; Rastogi et al., 2019; Beynel et al., 2020)
		Sphere	Mean	30 (13:17)	(Weise et al., 2022; BBB Zhang et al., 2022; Saturnino et al., 2021; Turi et al., 2022; Carlson et al., 2023; Caulfield and George, 2022; S Van Hoornweder et al., 2022; Mosayebi-Samani et al., 2021; KA Caulfield et al., 2020; KA Caulfield et al., 2022; Ciechanski et al., 2018; Dmochowski et al., 2013; Evans et al., 2020; Fekete et al., 2018; Klaus and Schutter, 2021; Liu et al., 2022; G Soleimani et al., 2022; S Van Hoornweder et al., 2022; Balderston et al., 2020; KA Caulfield et al., 2022; KA Caulfield et al., 2021; KA Caulfield et al., 2021; Janssen et al., 2014; Janssen et al., 2015; Ni et al., 2023; SV Van Hoornweder et al., 2021; S Van Hoornweder et al., 2022; Yamamoto et al., 2016; H Zhang et al., 2022)
			Percentile	8 (3:5)	(Weise et al., 2022; Saturnino et al., 2015; Hsu et al., 2023; Marquardt et al., 2021; Dmochowski et al., 2013; Janssen et al., 2015; Carla Piastra et al., 2021)
			Mode	1 (0:1)	(Jog et al., 2021)
		Hexa- hedron	Percentile	2 (0:2))	(Filmer et al., 2019; M Parazzini et al., 2017)
			Mean	2 (0:2)	(KA Caulfield et al., 2020; Galletta et al., 2015)
		Cylinder	Percentile	2 (0:2)	(SM Rampersad et al., 2014; Santos et al., 2016)
			Mean	1 (0:1)	(SM Rampersad et al., 2014)
E_┴, ‖_	ROI	Cylinder	Mean	1 (0:1)	(SM Rampersad et al., 2014)

|E| = magnitude of the electric field (E-Field), E_┴, ‖_ = normal or tangential components of the E-field, ROI = region of interest.

**Table 3:** Surface-level E-field outcome measures.

Component	Level		Type	Measure	N (TMS:tES)

|E|	Whole	Brain	Percentile	20 (6:14)	(Nandi et al., 2022; D Antonenko et al., 2021; SM Rampersad et al., 2014; Jiang et al., 2020; Bortoletto et al., 2016; Csifcsák et al., 2018; Cvetković et al., 2015; Cvetkovic et al., 2023; Kessler et al., 2013; Laakso et al., 2015; Metwally et al., 2015; Miranda et al., 2013; N Mittal et al., 2022; N Mittal et al., 2022; Zmeykina et al., 2020; D Antonenko et al., 2021; Antonenko et al., 2022; Im et al., 2019; Yuan et al., 2023; Can et al., 2019)
			Mean	4 (0:4)	(Nandi et al., 2022; Kalloch et al., 2022; Csifcsák et al., 2018; Miranda et al., 2013)
			Element	1 (0:1)	(Leaver et al., 2022)
	ROI	Structure	Percentile	17 (5:12)	(Edwards et al., 2013; Csifcsák et al., 2018; Laakso et al., 2015; Zmeykina et al., 2020; Im et al., 2019; Boayue et al., 2018; Borrione et al., 2020; Fujimoto et al., 2017; Gomez-Tames et al., 2020; J Gomez-Tames et al., 2019; Gomez-Tames et al., 2018; Hamajima et al., 2023; Konakanchi et al., 2020; Mikkonen and Laakso, 2019; Mikkonen et al., 2018; Pizem et al., 2022)
			Mean	14 (3:11)	(D Antonenko et al., 2021; Bhalerao et al., 2021; Csifcsák et al., 2018; Zmeykina et al., 2020; D Antonenko et al., 2021; Boayue et al., 2018; Borrione et al., 2020; Fujimoto et al., 2017; J Gomez-Tames et al., 2019; Hamajima et al., 2023; Mikkonen et al., 2018; Batsikadze et al., 2019; van der Burght et al., 2022; Kuhnke et al., 2020)
		Sphere	Mean	5 (1:4)	(Antonenko et al., 2019; Kalloch et al., 2022; Bortoletto et al., 2016; Lohse et al., 2020; Evans et al., 2022)
		Hexa-hedron	Mean	2 (1:1)	(Nandi et al., 2022; C Li et al., 2019)
			Percentile	1 (0:1)	(Nandi et al., 2022)
E_┴, ‖_	Whole	Brain	Percentile	12 (0:12)	(Nandi et al., 2022; Saturnino et al., 2017; Csifcsák et al., 2018; Metwally et al., 2015; Miranda et al., 2013; Splittgerber et al., 2020; Im et al., 2019; Yuan et al., 2023)
			Mean	5 (0:5)	(Nandi et al., 2022; Csifcsák et al., 2018; Miranda et al., 2013)
	ROI	Structure	Percentile	8 (1:7)	(Csifcsák et al., 2018; Zmeykina et al., 2020; Im et al., 2019; Hamajima et al., 2023; Callejon-Leblic and Miranda, 2019; Rasmussen et al., 2021)
			Mean	8 (1:7)	(Csifcsák et al., 2018; Zmeykina et al., 2020; Hamajima et al., 2023; Callejon-Leblic and Miranda, 2019; Laakso et al., 2016; Nawani et al., 2023; Splittgerber et al., 2021)
		Sphere	Mean	8 (4:4)	(BBB Zhang et al., 2022; Antonenko et al., 2019; Hsu et al., 2023; Janssen et al., 2014; Janssen et al., 2015; Evans et al., 2022)
			Percentile	1 (0:1)	(Janssen et al., 2015)
			Mode	1 (1:0)	(Jog et al., 2021)
		Hexa-hedron	Mean	2 (0:2)	(Nandi et al., 2022)
			Percentile	2 (0:2)	(Nandi et al., 2022)

|E| = magnitude of the electric field (E-Field), E_┴, ‖_ = normal or tangential components of the E-field, ROI = region of interest.

**Table 4:** Pros and cons of the most common electric field modeling outcome measures.

	Structure ROI	Geometric ROI	Whole brain percentile

Pros	Confined: Defining an ROI facilitates interpretation of E- field magnitudes within a predefined brain area. Highly flexible: The ROI can be tailored to available neuroimaging data and/or specific research questions Individualized: Size of the ROI is personalized to factors such as brain size and anatomical features.	Confined: Defining an ROI facilitates interpretation of E-field magnitudes within a predefined brain area. Flexible: The center of the ROI can position based neuroimaging data and/or research questions. Transferrable: Similar volumes across brain regions, participants or montages are analyzed.	Unconfined: By considering the whole brain, information is always given about the peak E-field magnitude regardless of location Reproducible: Easy to replicate as only a single percentile value is required to obtain the same results
Cons	Confined: By focusing on only one ROI, other important E-fields outside of the ROI may be overlooked. Transferrable: Uniqueness may hinder comparisons with other structural ROIs defined via different atlases or data.	Confined: By focusing on only one ROI, other important E-fields outside of the ROI may be overlooked. Size: Defining ROI size can be arbitrary, yet it can strongly affect the obtained E-field magnitude. Included region: Different cytoarchitectural / functional regions may be included in the same ROI across persons.	Spatially uncertain: Different brain volumes and regions may be analyzed across montages and participants. This can also impede interpretation of the obtained E-field magnitude.

E-Field = electric field, ROI = Region of Interest.

## Data Availability

The MRI-scans used for modeling are available via the original database (Human Connectome Project). The code used to obtain these results are obtained via motivated request to the corresponding authors.
