# Supplementary material for: Outcome measures for electric field modeling in tES and TMS: A systematic review and large-scale modeling study
Source: Neuroimage. Author manuscript; Available in PMC 2024 Apr 11. (PMC11008458; doi:10.1016/j.neuroimage.2023.120379)
Supplement: 1 [file NIHMS1936453-supplement-1.docx]

**Supplementary materials 1.** **Search keys**

| Database | Search Key | Results |
| --- | --- | --- |
| PubMed | ((((((electrical field[Title/Abstract]) OR (electrical fields[Title/Abstract])) OR (E-field[Title/Abstract])) OR (E-fields[Title/Abstract])) OR (electric field[Title/Abstract])) OR (electric fields[Title/Abstract])) AND (((((((((((noninvasive brain stimulation[Title/Abstract]) OR (non-invasive brain stimulation[Title/Abstract])) OR (non invasive brain stimulation[Title/Abstract])) OR (NIBS[Title/Abstract])) OR (tDCS[Title/Abstract])) OR (TMS[Title/Abstract])) OR (tACS[Title/Abstract])) OR (tES[Title/Abstract])) OR (transcranial magnetic stimulation[Title/Abstract])) OR (transcranial direct current stimulation[Title/Abstract])) OR (transcranial alternating current stimulation[Title/Abstract]))  < 10 years  Humans | 409 |
| Scopus | ( ( TITLE-ABS-KEY ( noninvasive AND brain AND stimulation ) OR TITLE-ABS-KEY ( nibs ) OR TITLE-ABS-KEY ( non-invasive AND brain AND stimulation ) OR TITLE-ABS-KEY ( tdcs ) OR TITLE-ABS-KEY ( tms ) OR TITLE-ABS-KEY ( tacs ) OR TITLE-ABS-KEY ( tes ) OR TITLE-ABS-KEY ( transcranial AND magnetic AND stimulation ) OR TITLE-ABS-KEY ( transcranial AND direct AND current AND stimulation ) OR TITLE-ABS-KEY ( transcranial AND alternating AND current AND stimulation ) OR TITLE-ABS-KEY ( non AND invasive AND brain AND stimulation ) ) AND PUBYEAR > 2012 AND PUBYEAR > 2012 ) AND ( ( TITLE-ABS-KEY ( electrical AND field ) OR TITLE-ABS-KEY ( electrical AND fields ) OR TITLE-ABS-KEY ( e-fields ) OR TITLE-ABS-KEY ( e-field ) OR TITLE-ABS-KEY ( electric AND field ) OR TITLE-ABS-KEY ( electric AND fields ) ) AND PUBYEAR > 2012 AND PUBYEAR > 2012 ) AND ( LIMIT-TO ( DOCTYPE,"ar" ) OR LIMIT-TO ( DOCTYPE,"cp" ) OR LIMIT-TO ( DOCTYPE,"re" ) OR LIMIT-TO ( DOCTYPE,"le" ) ) AND ( LIMIT-TO ( SUBJAREA,"NEUR" ) ) AND ( LIMIT-TO ( LANGUAGE,"English" ) ) | 767 |
| Web Of Science | ((((TS=(electric field)) OR TS=(electric fields)) OR TS=(E-field)) OR TS=(E-FIELDS)) OR TS=(electric fields)  ((((((((((TS=(noninvasive brain stimulation)) OR TS=(non-invasive brain stimulation)) OR TS=(non invasive brain stimulation)) OR TS=(NIBS)) OR TS=(tDCS)) OR TS=(TMS)) OR TS=(tACS)) OR TS=(tES)) OR TS=(transcranial magnetic stimulation)) OR TS=(transcranial direct current stimulation)) OR TS=(transcranial alternating current stimulation)  PY=(2013-2023)  LA=(English)  DT=(Review OR Article)  #1 AND #2 AND #3 AND #4 AND #5 | 1038 |

**Supplementary materials 2. Conductivity values used for electric field simulations**

| **Tissue Name** | **Value (S/m)** | **Tissue Name** | **Value (S/m)** |
| --- | --- | --- | --- |
| White Matter | 0.126 | Compact Bone | 0.008 |
| Gray Matter | 0.275 | Spongy Bone | 0.025 |
| CSF | 1.654 | Blood | 0.600 |
| Bone | 0.010 | Muscle | 0.160 |
| Scalp | 0.465 | Silicone Rubber | 29.400 |
| Eye balls | 0.500 | Saline | 1.000 |

**Supplementary materials 3. Identification of peak electric field value (V/m)**

**
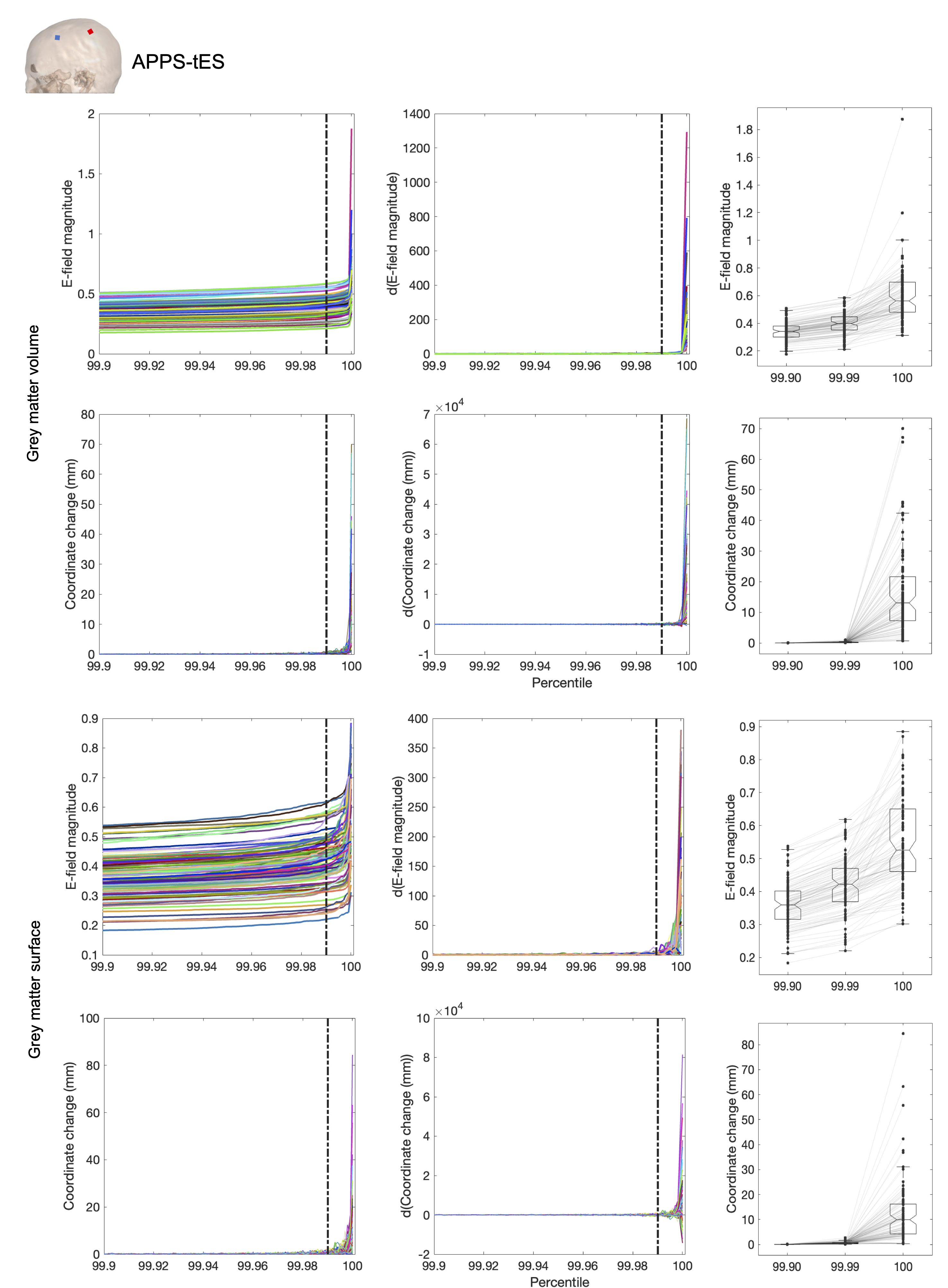
**

**Figure S3.1.** Evaluation of different electric field magnitude values and the associated 3D location across different percentiles used to approximate the peak electric field magnitude. P99.9 was withheld as peak electric field value due to it being the highest value that was stable both across the grey matter volume and surface, that was also used in several previous studies.

**
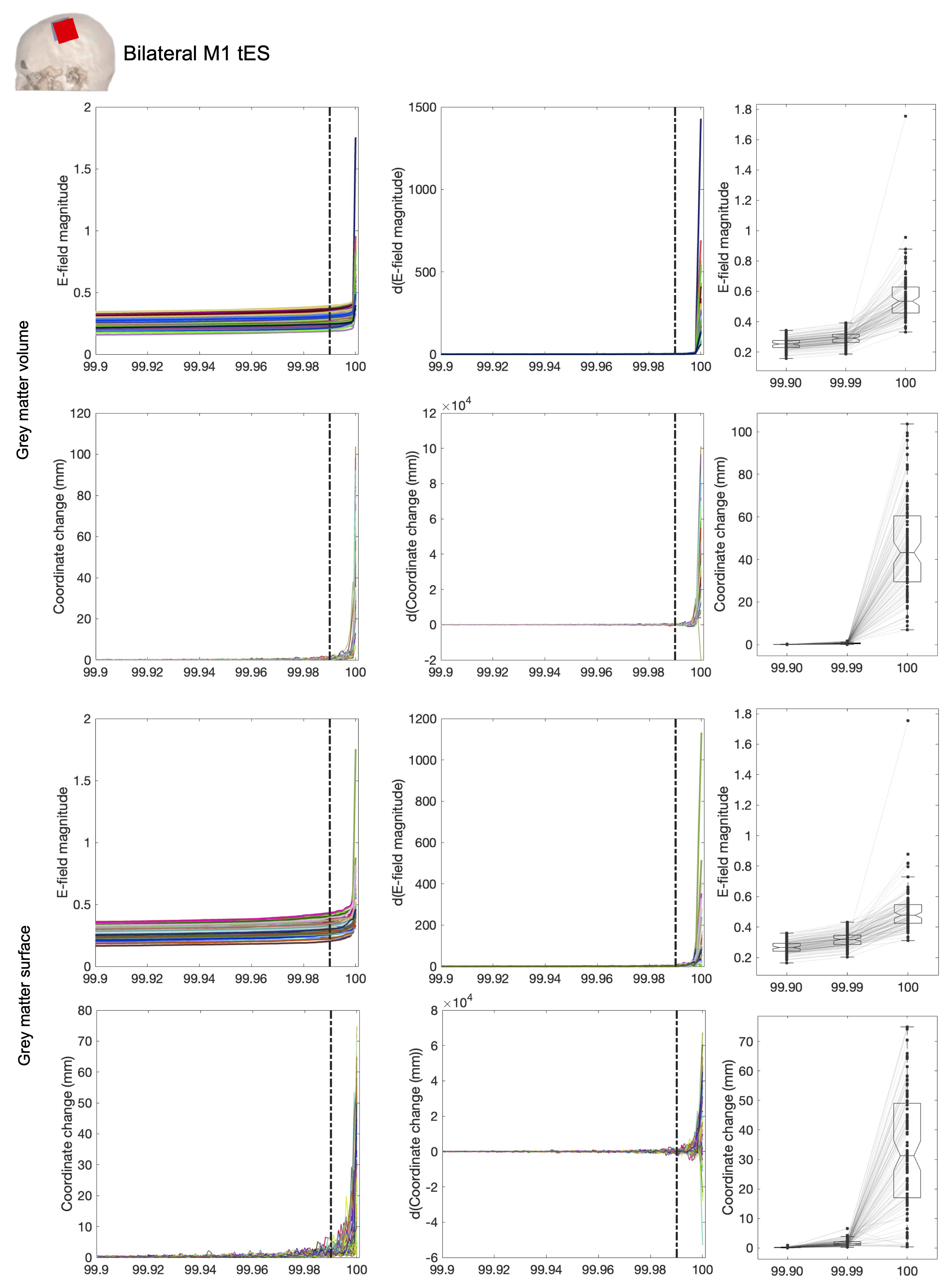
**

**Figure S3.2.** Evaluation of different electric field magnitude values and the associated 3D location across different percentiles used to approximate the peak electric field magnitude. P99.9 was withheld as peak electric field value due to it being the highest value that was stable both across the grey matter volume and surface, that was also used in several previous studies.

**
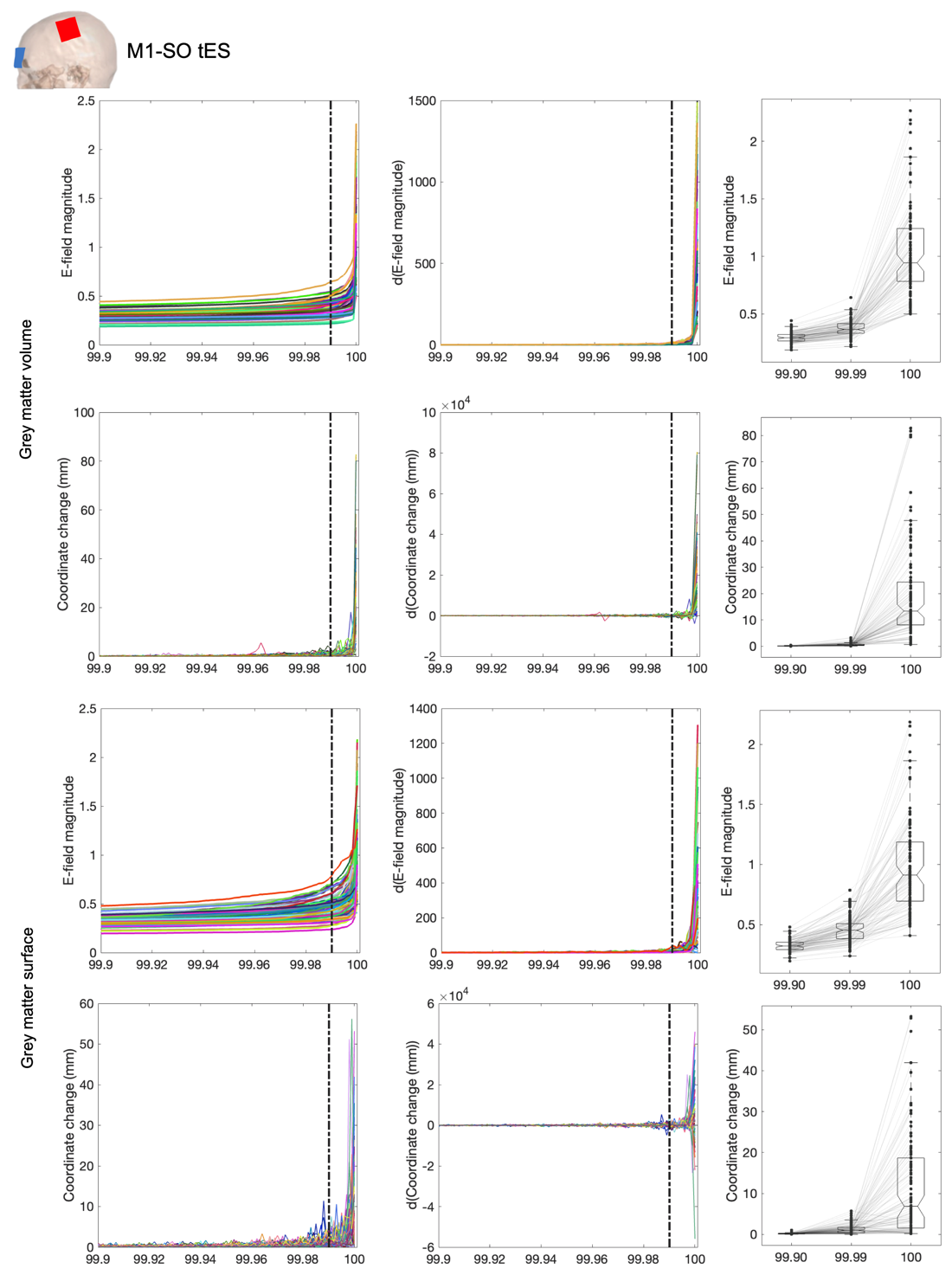
**

**Figure S3.3.** Evaluation of different electric field magnitude values and the associated 3D location across different percentiles used to approximate the peak electric field magnitude. P99.9 was withheld as peak electric field value due to it being the highest value that was stable both across the grey matter volume and surface, that was also used in several previous studies.

**
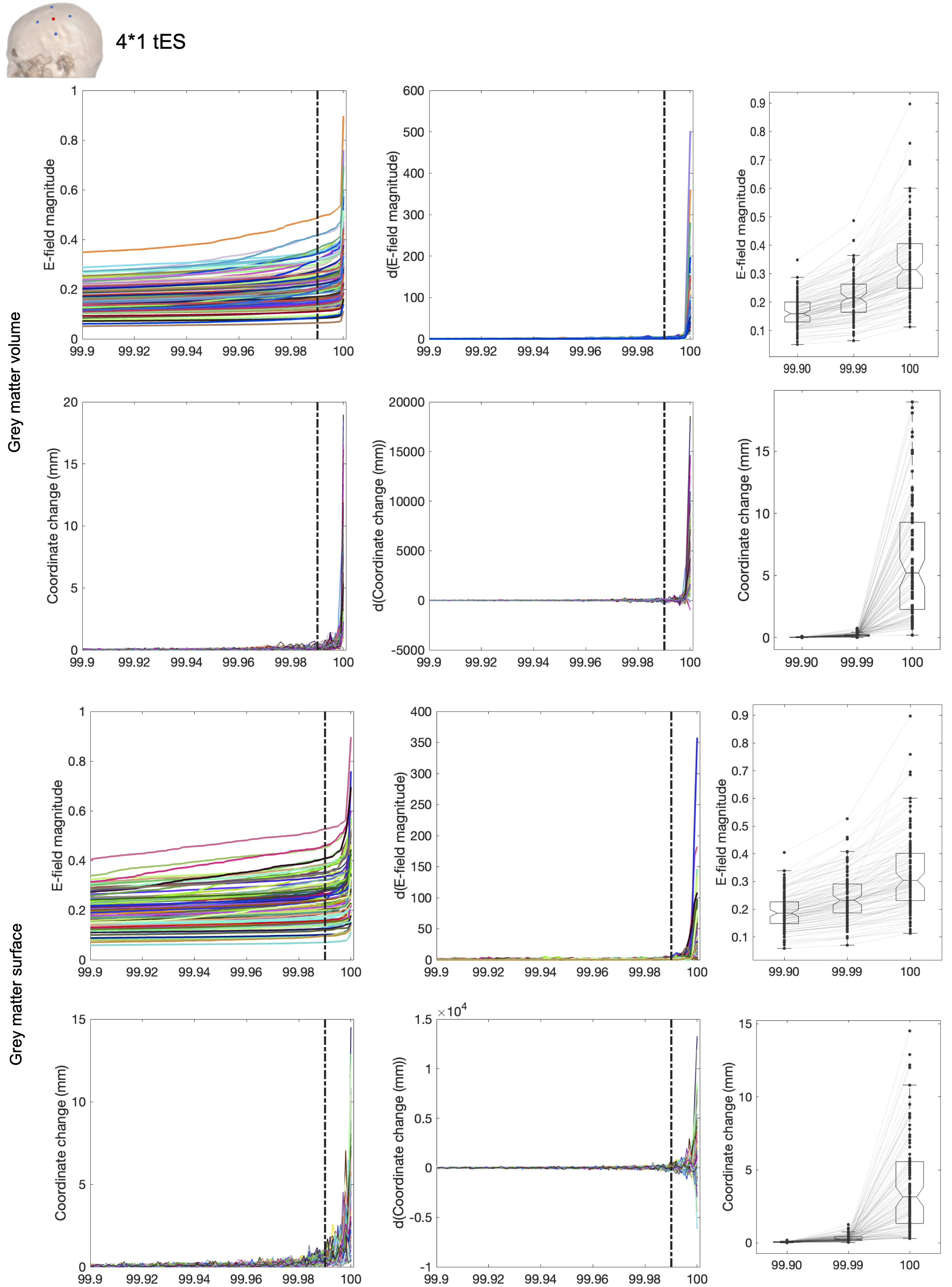
**

**Figure S3.4.** Evaluation of different electric field magnitude values and the associated 3D location across different percentiles used to approximate the peak electric field magnitude. P99.9 was withheld as peak electric field value due to it being the highest value that was stable both across the grey matter volume and surface, that was also used in several previous studies.

**
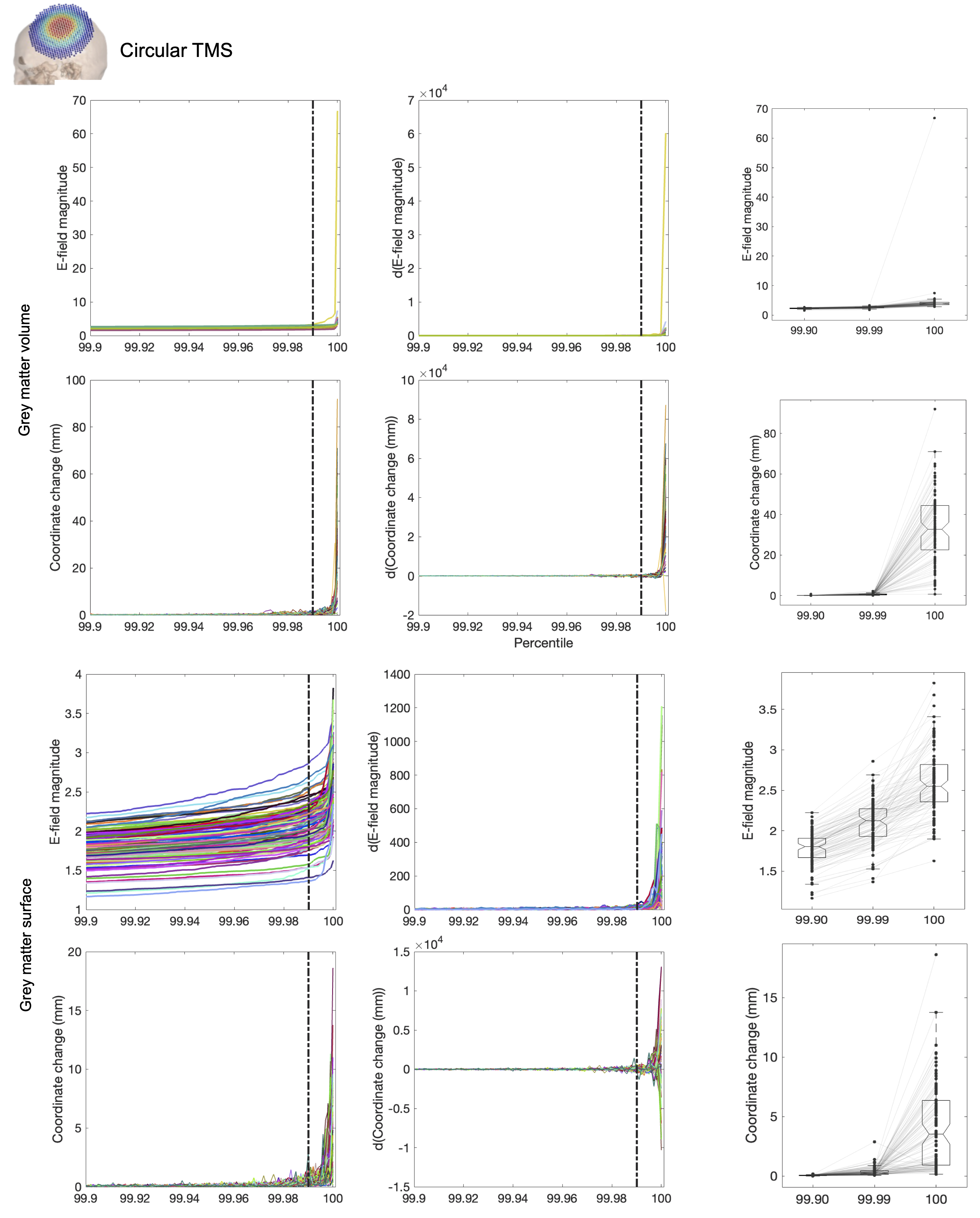
**

**Figure S3.5.** Evaluation of different electric field magnitude values and the associated 3D location across different percentiles used to approximate the peak electric field magnitude. P99.9 was withheld as peak electric field value due to it being the highest value that was stable both across the grey matter volume and surface, that was also used in several previous studies. Here, we show the results at a stimulation intensity of 1 dI/dt. Thus, these results can be easily scaled by multiplying with the wanted intensity.


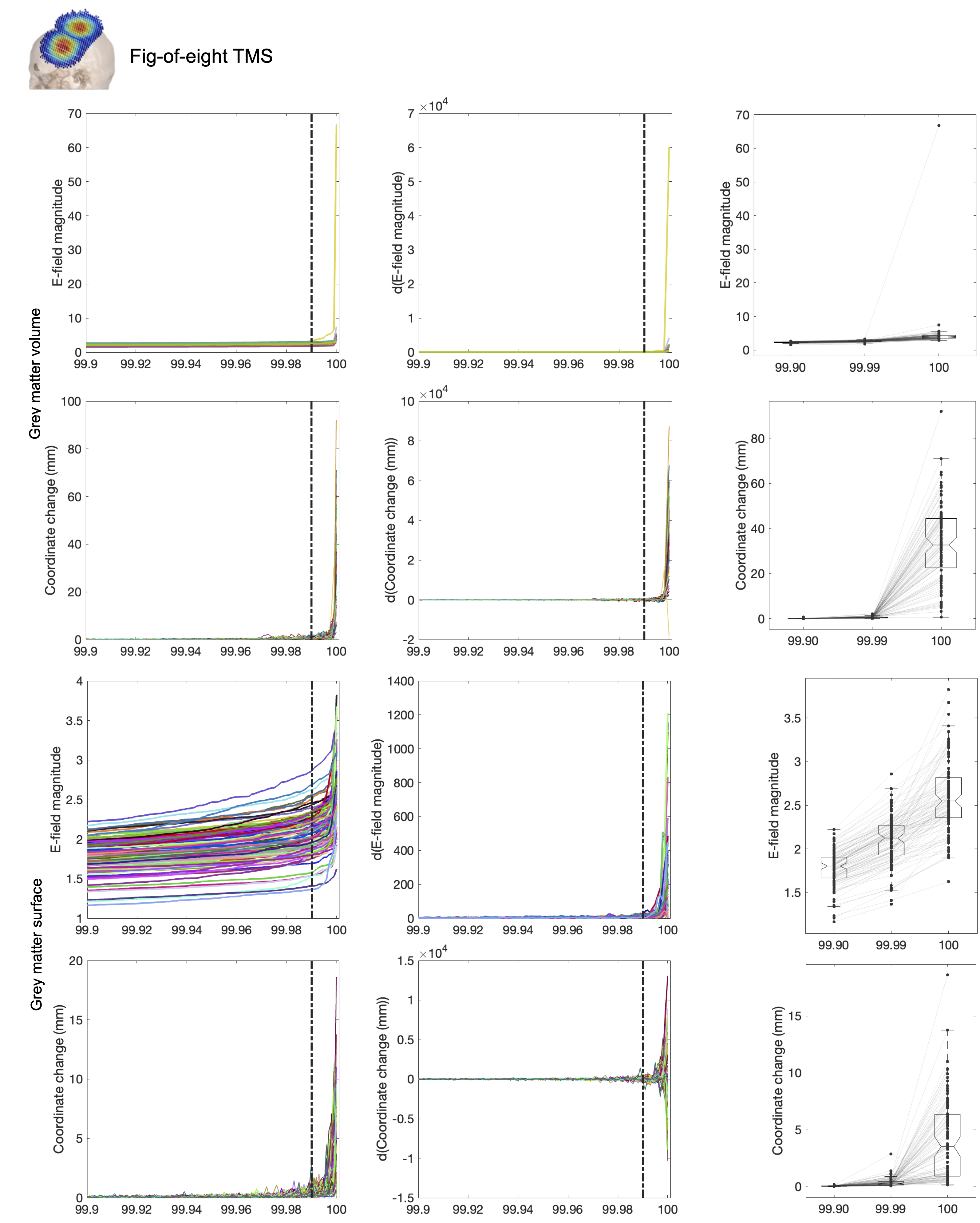


**Figure S3.6.** Evaluation of different electric field magnitude values and the associated 3D location across different percentiles used to approximate the peak electric field magnitude. P99.9 was withheld as peak electric field value due to it being the highest value that was stable both across the grey matter volume and surface, that was also used in several previous studies. Here, we show the results at a stimulation intensity of 1 dI/dt. Thus, these results can be easily scaled by multiplying with the wanted intensity.
